# Pathological Networking of Gray Matter Dendritic Density With Classic Brain Morphometries in OCD

**DOI:** 10.1001/jamanetworkopen.2023.43208

**Published:** 2023-11-13

**Authors:** Xiaochen Zhang, Jiajia Zhou, Yongjun Chen, Lei Guo, Zhi Yang, Trevor W. Robbins, Qing Fan

**Affiliations:** 1 Shanghai Mental Health Center, Shanghai Jiao Tong University School of Medicine, Shanghai, China; 2 Department of Psychology, University of Cambridge, Cambridge, United Kingdom; 3 Institute of Science and Technology for Brain-Inspired Intelligence, Fudan University, Shanghai, China; 4 Shanghai Key Laboratory of Psychotic Disorders, Shanghai, China; 5 Mental Health Branch, China Hospital Development Institute, Shanghai Jiao Tong University, Shanghai, China; 6 Now with Nanjing Brain Hospital Affiliated to Nanjing Medical University, Nanjing, China; 7 Now with Beijing Anding Hospital, Capital Medical University, Beijing, China

## Abstract

**Importance:**

The pathogenesis of obsessive-compulsive disorder (OCD) may involve altered dendritic morphology, but in vivo imaging of neurite morphology in OCD remains limited. Such changes must be interpreted functionally within the context of the multimodal neuroimaging approach to OCD.

**Objective:**

To examine whether dendritic morphology is altered in patients with OCD compared with healthy controls (HCs) and whether such alterations are associated with other brain structural metrics in pathological networks.

**Design, Setting, and Participants:**

This case-control study used cross-sectional data, including multimodal brain images and clinical symptom assessments, from 108 patients with OCD and 108 HCs from 2014 to 2017. Patients with OCD were recruited from Shanghai Mental Health Center, Shanghai, China, and HCs were recruited via advertisements. The OCD group comprised unmedicated adults with a *Diagnostic and Statistical Manual of Mental Disorders* (Fourth Edition) (*DSM-IV*) diagnosis of OCD, while the HCs were adults without any *DSM-IV* diagnosis, matched for age, sex, and education level. Data were analyzed from September 2019 to April 2023.

**Exposure:**

*DSM-IV* diagnosis of OCD.

**Main Outcomes and Measures:**

Multimodal brain imaging was used to compare neurite microstructure and classic morphometries between patients with OCD and HCs. The whole brain was searched to identify regions exhibiting altered morphology in patients with OCD and explore the interplay between the brain metrics representing these alterations. Brain-symptom correlations were analyzed, and the performance of different brain metric configurations were evaluated in distinguishing patients with OCD from HCs.

**Results:**

Among 108 HCs (median [IQR] age, 26 [23-31] years; 50 [46%] female) and 108 patients with OCD (median [IQR] age, 26 [24-31] years; 46 [43%] female), patients with OCD exhibited deficient neurite density in the right lateral occipitoparietal regions (peak *t* = 3.821; *P* ≤ .04). Classic morphometries also revealed widely-distributed alterations in the brain (peak *t* = 4.852; maximum *P* = .04), including the prefrontal, medial parietal, cingulate, and fusiform cortices. These brain metrics were interconnected into a pathological brain network associated with OCD symptoms (global strength: HCs, 0.253; patients with OCD, 0.941; *P* = .046; structural difference, 0.572; *P* < .001). Additionally, the neurite density index exhibited high discriminatory power in distinguishing patients with OCD from HCs (accuracy, ≤76.85%), and the entire pathological brain network also exhibited excellent discriminative classification properties (accuracy, ≤82.87%).

**Conclusions and Relevance:**

The findings of this case-control study underscore the utility of in vivo imaging of gray matter dendritic density in future OCD research and the development of neuroimaging-based biomarkers. They also endorse the concept of connectopathy, providing a potential framework for interpreting the associations among various OCD symptom–related morphological anomalies.

## Key Points

**Question:**

Is obsessive-compulsive disorder (OCD) associated with altered dendritic morphology and are such alterations associated with other brain structural metrics?

**Findings:**

In this case-control study including 108 patients with OCD matched with 108 healthy controls, patients with OCD exhibited deficient neurite density in the right lateral occipitoparietal regions, along with alterations in gray matter volume, thickness, gyrification, and white matter diffusivity in multiple cortical regions. These metrics formed a pathological brain network associated with OCD symptoms, supporting the concept of connectopathy that offers a potential framework for interpreting the association between morphological anomalies.

**Meaning:**

These findings suggest that in vivo imaging of gray matter dendritic density could serve as a valuable tool for understanding OCD and developing neuroimaging-based biomarkers.

## Teaser

This case-control study examines whether dendritic morphology is altered in patients with obsessive-compulsive disorder (OCD) compared with healthy controls and whether such alterations are associated with other brain structural metrics in pathological networks.

## Introduction

Obsessive-compulsive disorder (OCD) is a prevalent and debilitating mental disorder,^1^ affecting 2.3% of individuals in the US^2^ and 2.4% of individuals in China.^3^ Emerging evidence suggests that alterations in dendritic morphology, and thus synaptic plasticity, may contribute to the pathogenesis of OCD.^4^ However, in vivo neurite morphology imaging remains limited. Several OCD-associated genes, including *DLGAP1*, *DLGAP3*, and *SHANK3*, regulate dendritic spine morphology.^4,5^ Canine models exhibiting OCD-like behaviors have corroborated this link.^6^ In silico analysis of these genes has revealed proteins regulating postsynaptic dendritic spine formation, affecting dendritic spine morphology directly.^7^ Elevated plasma levels of microRNA-132 and microRNA-134 in patients with OCD suggest a potential impact on dendrite number and synapse formation in the cerebral cortex.^8^ A 2022 postmortem investigation identified a general decrease in dendritic spine density in the orbitofrontal cortex of patients with OCD.^9^ These findings suggest that neurite morphology, specifically gray matter dendritic morphology, is altered in OCD. Nevertheless, this hypothesis remains underexplored, leaving the degree and distribution of these alterations largely unknown. In this study, we used neurite orientation dispersion and density imaging (NODDI) to model microstructural features directly related to neuronal morphology, including nerve density and directional dispersion.^10^ While NODDI has revealed microstructural alterations in various psychiatric disorders,^11,12,13^ its application in OCD research has hitherto been unexplored, to our knowledge.

Previous studies using classic brain morphometries have identified widespread structural alterations across the brain in patients with OCD, including frontal, temporal, parietal, limbic, cerebellar, and striatal regions.^14,15,16,17^ However, these alterations, alongside changes in interregional connectivity,^18,19,20^ within or beyond the cortico-striatal-thalamo-cortical (CSTC) circuit,^21,22,23^ have not been holistically elucidated, thus impeding the interpretation of in vivo dendritic morphology imaging within the context of conventional brain metrics.

Thus, this investigation not only aimed to examine variations in gray matter dendritic morphology in OCD but also to explore their interplay with classic brain morphometries. Specifically, multimodal brain images were used to highlight microstructural alterations of neurite morphology and classic morphometric aberrations among patients with OCD. Voxel-wise analysis of microstructural measures derived from NODDI and skeletonized via gray matter-based spatial statistics (GBSS)^12,24^ was conducted to identify potential anomalies in gray matter neurite morphology in OCD. Additionally, classic morphometries identified widespread anomalies across the brain in patients with OCD. Subsequently, a post hoc comparison of the interrelationships among identified brain metrics was conducted between healthy individuals and patients with OCD via a network approach. Associations with clinical symptoms were also explored. Different combinations of brain metrics were further evaluated for distinguishing patients with OCD from healthy individuals to explore the development of neuroimaging-based biomarkers.

## Methods

This case-control study was approved by the ethics committee of Shanghai Mental Health Center. All participants provided written informed consent. We followed the Strengthening the Reporting of Observational Studies in Epidemiology (STROBE) reporting guideline for observational studies.

### Participants

Patients with OCD were recruited from the outpatient clinic of Shanghai Mental Health Center, were assessed using the Mini International Neuropsychiatric Interview by psychiatrists, and completed the Yale-Brown Obsessive Compulsive Scale (Y-BOCS) to confirm a *Diagnostic and Statistical Manual of Mental Disorders* (Fourth Edition) (*DSM-IV*) diagnosis of OCD with minimal comorbidity. Healthy controls (HCs) were recruited via advertisements and were assessed by a trained psychiatrist to exclude *DSM-IV* diagnoses of any psychiatric disorder (eMethods 1 in Supplement 1). All participants completed the Hamilton Anxiety Rating Scale, 24-item Hamilton Depression Rating Scale, and State-Trait Anxiety Inventory to assess anxiety and depression levels.

### Statistical Analysis

#### Identifying OCD-Related Brain Alterations

Multimodal neuroimaging data were acquired from a Simens VERIO 3T scanner (eMethods 2 in Supplement 1) and analyzed per modality^25^ (eFigure 1 in Supplement 1). Multishell diffusion-weighted images (*b* = 1000 s/mm^2^ and *b* = 2000 s/mm^2^) were used to assess neurite morphology via the NODDI Matlab Toolbox^10^ in Matlab version R2019b (MathWorks), which derived neurite density index (NDI) and orientation dispersion index maps that were subsequently skeletonized via the NODDI GM-based spatial statistics (NODDI-GBSS)^24^ in FMRIB Software Library version 5.0.10 (eMethods 3 in Supplement 1). Additionally, conventional diffusion tensor imaging (DTI) measures,^26^ including fractional anisotropy, mean diffusivity (MD), axial diffusivity (AD), and radial diffusivity, were derived from the single-shell diffusion-weighted images (*b* = 1000 s/mm^2^) via tensor fitting and were subsequently skeletonized via the Tract-Based Spatial Statistics (TBSS)^27,28^ in FMRIB Software Library (eMethods 4 in Supplement 1). T1-weighted brain images underwent voxel-based morphometry^29^ for gray matter probability maps and surface-based morphometry for morphological metrics, including cortical thickness,^30^ gyrification,^31^ complexity,^32^ and sulcal depth^33^ (eMethods 5 in Supplement 1), using the CAT12 and SPM12 toolboxes in Matlab.

To address inconsistent literature and minimize preconceived bias, we used whole-brain analyses to identify OCD-associated brain structural alterations (eMethods 6 in Supplement 1). To test whether the diagnostic groups differed while controlling for age, sex, and education, the skeletonized parameter maps derived from the diffusion-weighted images underwent nonparametric voxelwise permutation analyses using the randomize function of FMRIB Software Library, and the parameter maps derived from the T1-weighted images underwent parametric voxelwise or vertexwise analyses in SPM12, with multiple comparisons corrected to control type I errors. Moreover, we examined whether the anatomical connectivity^34,35^ to the morphologically altered brain regions was impaired, and, if so, whether such impairments were mediated^36,37^ by the corresponding morphological metrics (eMethods 7 in Supplement 1).

#### Post Hoc Construction and Analyses of Brain Metric Networks

To explore the interplay between dendritic and other brain alterations associated with OCD, we compared the intermetric associations between groups through network analysis.^38^ The previously identified brain metrics served as nodes of the network and the partial correlations between them as edges. Specifically, we constructed 2 separate networks of brain morphological metrics (eMethods 8 in Supplement 1), one for either diagnostic group, and compared their edge weight stability, global strength, and network structure^39^ to assess network structural disparities (eMethods 9 in Supplement 1), using bootnet version 1.5 and NetworkComparisonTest version 2.2.1 packages in RStudio version 1.3 (Posit Software).

#### Post Hoc Correlational Analyses With Symptoms

We used Spearman rank correlation to assess brain-symptom correlations among patients with OCD. To investigate whether the collective information of all brain metrics indicating morphological alterations was symptomatically associated, we applied unsupervised hierarchical clustering to group the patients with OCD based solely on their brain data and explored potential discrepancies in Y-BOCS total scores among these subgroups (eMethods 10 in Supplement 1). These analyses were performed with SPSS statistical software version 26 (IBM). All statistical tests upheld a significance threshold of *P* < .05, with both unadjusted and adjusted *P* values for multiple comparisons.

#### Post Hoc Machine Learning–Based OCD vs HC Classification

We investigated the performance of various combinations of brain metrics in discriminating patients with OCD from healthy individuals (eMethods 11 in Supplement 1). We assessed the performance of each brain metric as the single input variable, followed by evaluating 4 combinations of the brain metrics obtained from the 4 metric-deriving procedures (NODDI + GBSS, DTI + TBSS, voxel-based morphometry, and surface-based morphometry), 2 combinations of brain metrics from the 2 image modalities (diffusion and T1), and a combination of all previously identified brain metrics. Logistic regression–based and support vector machine–based classifiers were used. These analyses were conducted in Matlab. Data were analyzed from September 2019 to April 2023.

## Results

### Demographic Characteristics

This study included 108 unmedicated patients with OCD (median [IQR] age, 26 [24-31] years; 46 [43%] female) and 108 HCs (median [IQR] age, 26 [23-31]; 50 [46%] female) matched for age, sex, and education level (Table). The 2 diagnostic groups were demographically matched (Table; eAppendix 1 in Supplement 1). The median (IQR) education level was 16 (15-17) years for HCs and 16 (15-16) years for patients with OCD.

**Table.** Demographic and Psychopathological Characteristics of the Participants

Characteristics	Participants, No. (%)	
	HC (n = 108)	OCD (n = 108)
Sex		
Male	58 (54)	62 (57)
Female	50 (46)	46 (43)
Age, median (IQR), y	26 (23-31)	26 (24-31)
Education, median (IQR), y	16 (15-17)	16 (15-16)
Y-BOCS		
Obsession, median (IQR), points	NA	14 (11-16)
Compulsion, median (IQR), points	NA	10 (9-14)
Total, median (IQR), points	NA	24 (20-29)
HARS, median (IQR), points^a^	0 (0-2)^b^	8 (5-12)^c^
HDRS, median (IQR), points^a^	0 (0-3)^d^	11 (8-18)^c^
STAI		
State anxiety, median (IQR), points^a^	33 (30-42)^b^	49 (42-57)^e^
Trait anxiety, median (IQR), points^a^	37 (34-44)^b^	55 (48-61)^d^

Abbreviations: HARS, Hamilton Anxiety Rating Scale; HC, healthy control; HDRS, Hamilton Depression Rating Scale; NA, not applicable; OCD, obsessive-compulsive disorder; STAI, State-Trait Anxiety Inventory; Y-BOCS, Yale-Brown Obsessive Compulsive Scale.

^a^ Between-group *P* < .001, after adjusting for age, sex, and education.

^b^ Data from 10 participants were unavailable.

^c^ Data from 1 participant were unavailable.

^d^ Data from 11 participants were unavailable.

^e^ Data from 13 participants were unavailable.

### OCD-Associated Alterations of Gray Matter Neurite Microstructure

**Figure 1.**
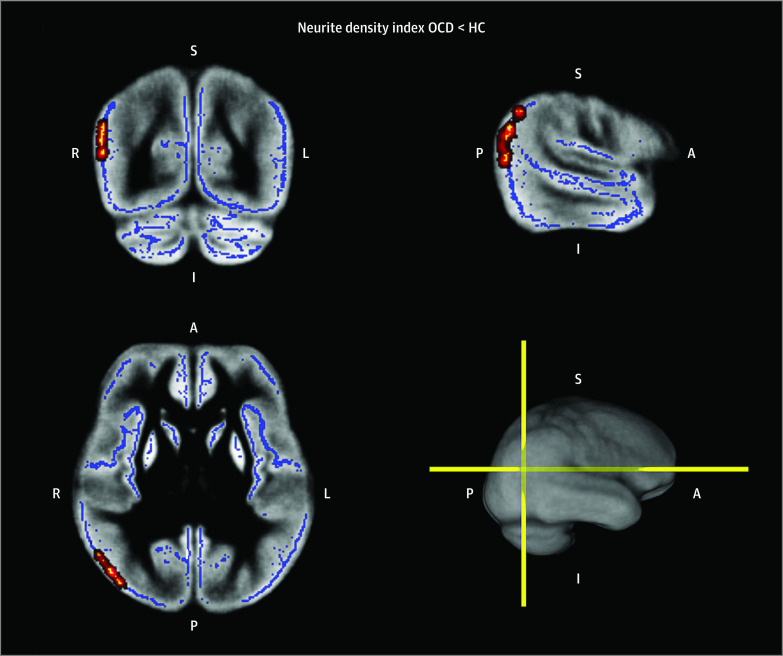
Results of the Neurite Orientation Dispersion and Density Imaging With Gray Matter-Based Spatial Statistics Analysis Patients diagnosed with obsessive-compulsive disorder (OCD) manifested deficient neurite density compared with healthy controls (HCs), specifically in the superior (S) section of the right (R) lateral occipital cortex and the right angular gyrus, extending to the posterior (P) division of the R supramarginal gyrus. The blue trace refers to the gray matter skeleton, and the red-yellow coloration, with brighter shades indicating significant alterations in the highlighted gray matter skeleton voxels and darker shades delineating an imaginary wrapper for visual emphasis, serves purely for visualization purposes and does not indicate alteration in severity. A indicates anterior; I, inferior; and L, left.

We discovered significantly lower NDI among patients with OCD compared with HCs (Figure 1; eFigure 2 and eAppendix 2 in Supplement 1), specifically in the superior section of the right lateral occipital cortex (peak *t* = 3.821; corrected *P* = .03) and the right angular gyrus (peak *t* = 3.446; corrected *P* = .03), extending to the posterior division of the right supramarginal gyrus (peak *t* = 2.292; corrected *P* = .04). Furthermore, patients with OCD showed deficient degree centrality of the right lateral occipital and inferior parietal cortices after adjusting for age, sex, and education (F_1,211_ = 4.245; *P* = .04; η^2^ = 0.020), indicating significantly impaired anatomical connectivity linking these 2 regions to others. This anatomical connectivity alteration was completely mediated by the local NDI (eFigure 2 in Supplement 1).

### OCD-Associated Alterations Revealed by Classic Morphometries

**Figure 2.**
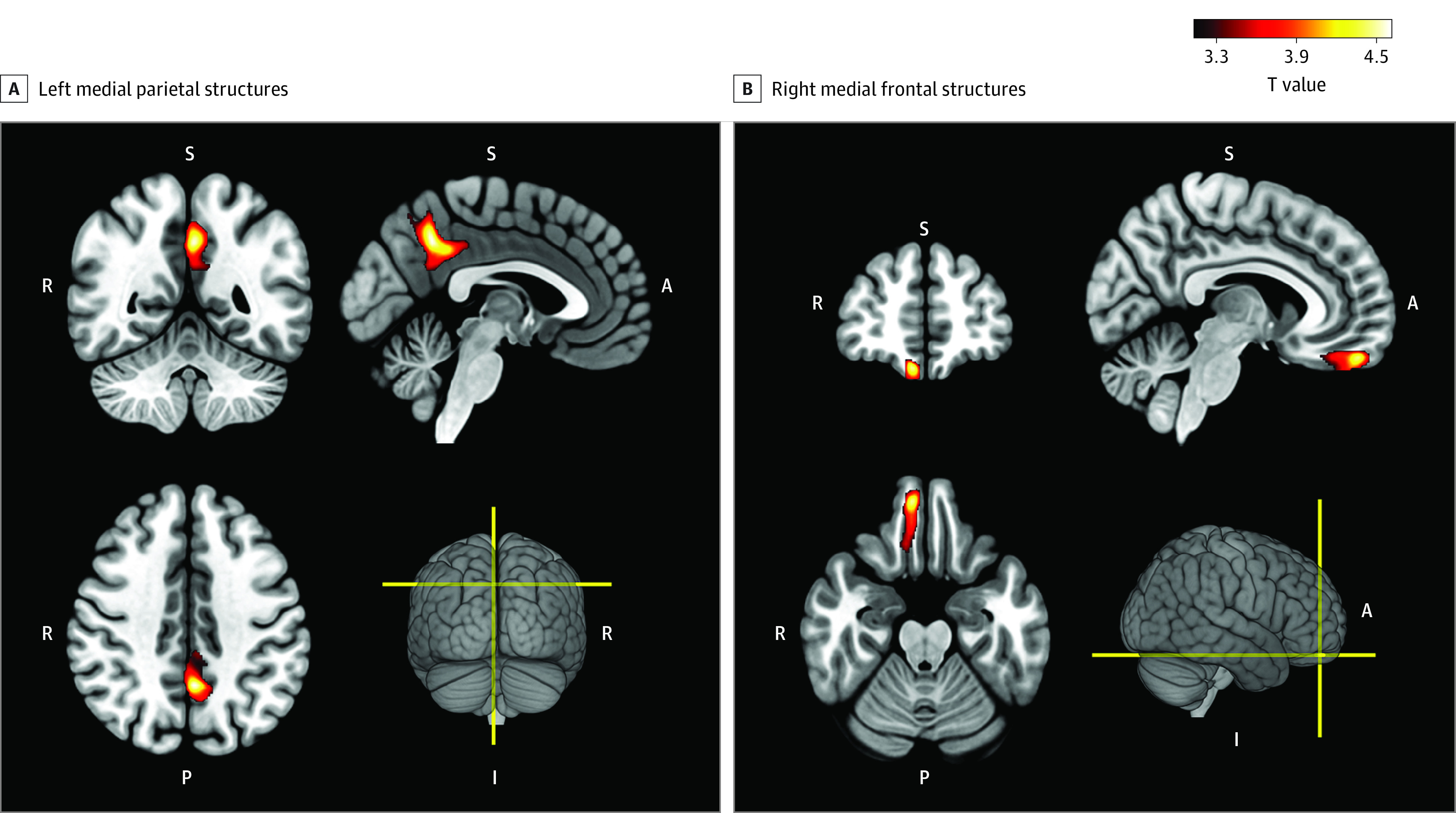
Results of the Voxel-Based Morphometry Compared with healthy controls patients with obsessive compulsive disorder displayed significantly deficient gray matter volume in (A) the left (L) medial parietal structures, ie, the precuneus and the posterior cingulate gyrus, and (B) right (R) medial frontal structures, ie, the medial orbital gyrus and the gyrus rectus. A indicates anterior; I, inferior; P, posterior; S, superior.

Compared with HCs, patients with OCD displayed significantly deficient gray matter volume in the left medial parietal structures (ie, precuneus and posterior cingulate gyrus; peak *t* = 4.62; corrected *P* = .001) (Figure 2A) and right medial frontal structures (ie, medial orbital gyrus and gyrus rectus; peak *t* = 4.49; corrected *P* = .03); (Figure 2B; eFigure 3, eTable 1, and eAppendix 3 in Supplement 1). In addition, patients with OCD demonstrated significantly deficient cortical thickness in the left fusiform gyrus (peak *t* = 4.35; corrected *P* = .04) (eTable 2 in Supplement 1) and disrupted local gyrification in right lateral frontal structures (ie, right superior and middle frontal gyri; peak *t* = 3.73, corrected *P* = .008) and left middle cingulate gyrus (peak *t* = 3.61; corrected *P* = .02) (eFigure 4 in Supplement 1). Interestingly, the degree centrality of the brain regions exhibiting gray matter morphological anomalies was not significantly altered among patients with OCD.

Regarding white matter, we identified an extensive area of significantly greater white matter AD (peak *t* = 4.852; corrected *P* = .006) and MD (right-lateralized; peak *t* = 4.797; corrected *P* = .03) among patients with OCD compared with HCs (eFigure 5 and eAppendix 4 in Supplement 1). Furthermore, the global strength of anatomical connectivity between all possible pairs of cortical regions among patients with OCD was significantly impaired after controlling for age, sex, and education (F_1,211_ = 5.235; *P* = .02; η^2^ = 0.024). This impairment was partially mediated by the AD and MD in the highlighted regions serially (eFigure 5 in Supplement 1).

### Emergence of Pathological Brain Network Among Patients With OCD

**Figure 3.**
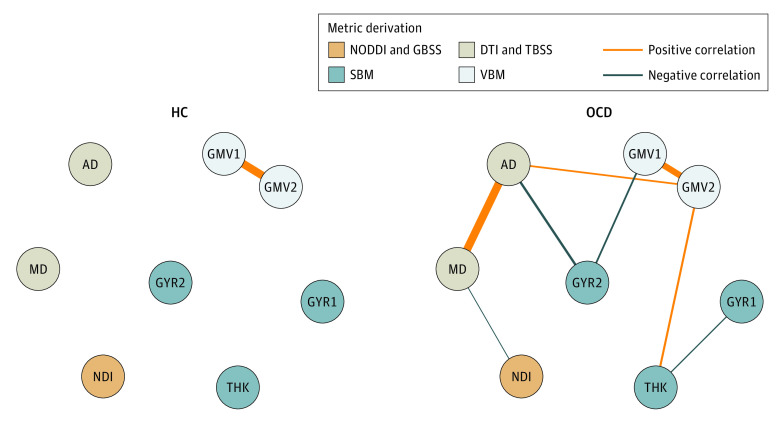
Results of the Network Analysis GMV1 and GMV2 refer to the gray matter volume values within the left medial parietal cluster and the right medial frontal cluster (ie, cluster 1 and 2, as defined in eTable 1 in Supplement 1), respectively. THK refers to the cortical thickness value within the left fusiform cluster (ie, cluster thickness 1, as defined in eTable 2 in Supplement 1). GYR1 and GYR2 refer to the gyrification values within the right lateral frontal cluster and the left middle cingulate cluster (ie, cluster gyrification 1 and gyrification 2, as defined in eTable 2 in Supplement 1), respectively. Thickness of lines indicates strength of correlation. AD indicates axial diffusivity; DTI, diffusion tensor imaging; GBSS, gray matter-based spatial statistics; HC, healthy control; MD, mean diffusivity; NDI, neurite density index; NODDI, neurite orientation dispersion and density imaging; OCD, obsessive-compulsive disorder; SBM, surface-based morphometry; TBSS, Tract-Based Spatial Statistics; VBM, voxel-based morphometry

Notable differences were observed in the intermetric connections between HCs and patients with OCD (Figure 3). In HCs, the only positive correlation was observed between gray matter volume indicators, with other intermetric associations absent. Conversely, among patients with OCD, all brain metrics were correlated with at least 1 other metric. Furthermore, a substantial difference was seen in the correlation stability coefficient of the brain metric network between groups, with a coefficient of 0.593 for patients with OCD, indicating a stable network, but a coefficient of 0.046 for HCs, indicating an unstable network. Additionally, a higher global strength of the brain metric network in patients with OCD (sum of all edge weights: HCs, 0.253; patients with OCD, 0.941; *P* = .046) and a significant network structural difference between groups (maximum edge difference, 0.572; *P* < .001) were found. These findings suggest that certain dissociated brain metrics in HCs were pathologically connected among patients with OCD, indicating the emergence of a pathological brain network unique to patients with OCD (eAppendix 5 in Supplement 1).

### Brain-Symptom Correlations

The brain alterations of a larger extent were generally correlated with more severe symptoms (of 6 significant correlations, 2 survived multiple comparison corrections) (eAppendix 6 and eFigure 6 in Supplement 1). Moreover, all 8 nodes of the pathological brain network together effectively differentiated patients with greater overall severity from those with milder overall symptoms, as measured by the Y-BOCS total score. These results collectively indicate an association between neurological phenomena and symptoms.

### Performance of Pathological Brain Network in Identifying Patients With OCD

The NDI at the right occipitoparietal regions outperformed all other brain metrics in classification accuracy, area under the curve values, and sensitivity, and ranked second in specificity (eFigure 7, eTable 3, and eAppendix 7 in Supplement 1). Additionally, the node derived from NODDI + GBSS outperformed those derived from DTI + TBSS (eTable 4 in Supplement 1). Combining these nodes yielded better classification than combining those from T1 images. Notably, the node derived from NODDI + GBSS outperformed all nodes derived from T1 images combined.

## Discussion

This case-control study used multishell diffusion images to explore gray matter dendritic morphology in an innovative approach to neuroimaging changes in OCD. Our primary findings showed a pronounced deficit in neurite density in the right lateral occipitoparietal regions among patients with OCD that completely mediated the impaired anatomical connectivity linking local regions to others. These findings offer valuable insights into the neural basis of OCD, and the high discriminative power of the neurite density index in differentiating patients with OCD from HCs highlights the significance of in vivo gray matter dendritic density imaging. The dendritic density, along with metrics displaying widespread morphological anomalies across the brain, demonstrated interconnections among patients with OCD but not among HCs, indicating the presence of a pathological brain network for OCD. Importantly, different nodes of this network were linked to various comorbid symptoms. Moreover, the complete pathological brain network provided relevant information for assessing the overall severity of OCD, showing the potential of the pathological brain network approach for OCD research and the development of neuroimaging-based biomarkers for OCD.

This may be the first study to examine microstructural changes in dendritic morphology associated with OCD. Among patients with OCD, we identified deficient neurite density in the right occipitoparietal regions. As dendrites are the primary constituents of neurites in gray matter, we infer that patients with OCD exhibit deficient dendritic density in these regions. Functionally, these regions are part of the occipitoparietal circuit of the dorsal visual pathway, crucial in spatial perception and the formation of egocentric frames of reference.^40^ Indeed, patients with OCD exhibit deficits in spatial information memory^41,42^ and visual-spatial perception,^43^ consistent findings also observed among adolescent patients with OCD.^44^ Recent research also has reported decreased intrinsic functional connectivity within the dorsal visual pathway^45^ or the lateral visual network^46^ in OCD but increased functional connectivity between the right superior lateral occipital cortex and the left lateral parietal cortex, which was positively correlated with symptom severity.^47^ Additionally, selective serotonin reuptake inhibitor treatment increases metabolic activity in the right superior occipital cortex with corresponding symptomatic and neuropsychological improvements.^48^ Increased provocation-induced activation^49^ and reduced gray matter volume^50,51,52^ have also been documented in these areas. Given the close association between dendritic morphology and synaptic plasticity, we found that the deficient dendritic density in the right lateral occipitoparietal regions completely mediated the disruption of anatomical connectivity from local to elsewhere. These results highlight the neural changes potentially impacting functioning in OCD, serving as a bridge connecting structural and functional brain anomalies in OCD and warranting further investigations into the association of deficient dendritic density with brain functional and cognitive anomalies.

Patients with OCD are often claimed to have anomalies in CSTC circuits,^53^ including striatum, thalamus, prefrontal cortex, and anterior cingulate cortex.^54^ Alterations are observed in various CSTC aspects, including resting-state functional connectivity^55^ and magnetic resonance imaging (MRI) spectroscopy.^56,57^ Some of our observations support this theory, revealing disrupted gyrification in right frontal and left cingulate regions, alongside deficient gray matter volume in right medial frontal regions. This region is implicated in cognitive inflexibility in OCD, indicated by more errors in attentional set-shifting.^20^ However, accumulating evidence, including the ENIGMA OCD consortium, suggests OCD-related brain alterations beyond frontostriatal circuits.^21,22,23,58^ Some of our results align with this, showing deficient neurite density in right inferior parietal cortex, consistent with the ENIGMA consortium’s report of thinned inferior parietal cortex among adult and pediatric patients with OCD^59^ and an Adolescent Brain Cognitive Development study of children with repetitive thoughts and actions implicating changes in the inferior parietal cortex and precuneus.^60^

The importance of altered brain network connectivity for psychiatric disorders^61^ is increasingly recognized. Our study found widespread white matter anomalies, evidenced by greater AD and MD, in patients with OCD. These results align with previous findings,^21^ although we found no significant changes in fractional anisotropy, suggesting possible axonal swelling without substantial axonal damage in OCD. Previous studies investigating white matter morphology have reported mixed DTI findings,^21,62^ reflecting the heterogeneity of OCD,^63^ although also supporting the concept of connectopathy.^64^ Crucially, our identification of morphological alterations in regions without apparent anatomical connectivity impairment implies connectopathy mechanisms beyond deficits simply in anatomical connectivity.

To further investigate neural substrates of OCD from a connectopathy perspective, we analyzed covarying associations among brain metrics displaying morphological alterations, regardless of the metric deriving methods (eAppendix 8 in Supplement 1). The covariance among the morphological metrics reflected the coordination of brain morphology shaped by genetic and environmental influences.^65^ Our study discovered that the brain metrics displaying neurite or macroscopic morphological alterations in patients with OCD were disconnected in healthy participants but were connected in patients with OCD. This covariance pattern deviates from the typical form of connectopathy with disrupted interregional connectivity^66,67^; thus, this pathological brain network is a distinctive feature of OCD, aligning with the concept of connectopathy and shedding light on latent pathological or etiological factors coordinating specific brain metrics for patients with OCD. Moreover, some nodes of this pathological network were correlated with patients’ symptoms, suggesting a potential parallel to the symptom network for OCD.^68^

We further explored the potential of brain morphological metrics as a neuroimaging-based biomarker for OCD. Our findings show that the dendritic density at right occipitoparietal regions is a promising feature for classifying patients with OCD and HCs, outperforming other individual metrics. Additionally, the brain metrics derived from diffusion-weighted images were more effective than those derived from T1-weighted images for HC vs OCD classification, consistent with prior research.^69^ Comparing our results with prior similar-sized single-site studies, the brain metrics presented in this study exhibit comparable or even enhanced classification performance.^70^ Note that some studies have reported better performance,^71,72^ possibly influenced by medication or imaging modality. While our preliminary findings suggest the potential utility of these brain metrics, particularly gray matter dendritic density, further validation studies using independent data sets and feature selection procedures are warranted.

### Limitations

Several limitations should be considered. First, the included patients with OCD were medication-free for at least 8 weeks before brain scanning, but we cannot completely rule out their prior use of psychiatric medications. As previous medication likely affects brain morphology and the performance of HC vs OCD classification,^70,73^ future studies with exclusively patients who are drug naive would be beneficial. Second, our data were cross-sectional. Longitudinal data would be invaluable for elucidating the roles of the identified dendritic changes in OCD pathogenesis. Third, this study only included adult patients. Since OCD is sometimes considered a neurodevelopmental disorder, future investigations into gray matter dendritic density alterations in pediatric patients are warranted. Fourth, although our network analysis assessed statistical outcomes independently of input data selection criteria,^74^ it might still be susceptible to the selective inference problem.^75^ Nevertheless, our findings based on post hoc analyses still present well-substantiated hypotheses awaiting further validation in independent data sets. Fifth, the machine learning–based HC vs OCD classification using brain metrics may overestimate its performance. Future studies should use more rigorous validation procedures in independent data sets to systematically assess the potential biomarker candidates.

## Conclusions

This case-control study found deficient neurite density in the right lateral occipitoparietal regions of patients with OCD, a robust discriminator of OCD, compared with healthy individuals. It highlights the potential of in vivo gray matter dendritic morphology imaging in future OCD research and biomarker development. Moreover, the brain metrics indicating OCD-associated neurite and macroscopic morphological alterations constituted a symptomatically related pathological brain network in OCD, providing a framework for interpreting the associations between various morphological abnormalities.

## Notes

Supplement 1. **eMethods 1.** Eligibility and Exclusion Criteria **eMethods 2.** Magnetic Resonance Image Acquisition **eMethods 3.** Processing of Multishell Diffusion-Weighted MR Images **eMethods 4.** Processing of Single-Shell Diffusion-Weighted MR Images **eMethods 5.** Processing of T1 Images **eMethods 6.** Whole Brain Analyses of Morphological Metrics **eMethods 7.** Post Hoc Anatomical Connectivity Analyses **eMethods 8.** Post Hoc Construction of Networks of Brain Metrics **eMethods 9.** Post Hoc Analyses of Networks of Brain Metrics **eMethods 10.** Post Hoc Correlational Analyses With Symptoms **eMethods 11.** Post Hoc Machine Learning-Based OCD vs HC Classification **eAppendix 1.** Demographic Characteristics **eAppendix 2.** OCD-Associated Alterations of Gray Matter Microstructure **eAppendix 3.** OCD-Associated Alterations of Gray Matter Morphology **eAppendix 4.** OCD-Associated Alterations of White Matter Morphology and Microstructure **eAppendix 5.** Emergence of a Pathological Brain Network Among Patients With OCD **eAppendix 6.** Correlations Between Nodes of OCD Pathological Brain Network and Clinical Symptoms **eAppendix 7.** Performance of Pathological Brain Network in Identifying OCD Patients **eAppendix 8.** Supplemental Discussion **eTable 1.** Gray Matter Volume Alterations in Patients With OCD **eTable 2.** Gray Matter Surface Morphological Alterations in Patients With OCD **eTable 3.** Performance Comparison of Brain Metrics for OCD Patient Identification **eTable 4.** Performance Comparison of Brain Metric Combinations for OCD Patient Identification **eFigure 1.** The Complete Workflow for Analyzing Neuroimaging Data **eFigure 2.** Results of the NODDI-GBSS Analysis **eFigure 3.** Results of the Voxel-Based Morphometry **eFigure 4.** Results of the Surface-Based Morphometries **eFigure 5.** Results of the White Matter TBSS Analysis **eFigure 6.** Correlations Between Clinical Symptoms and Nodes of the Pathological Brain Network in OCD Patients **eFigure 7.** Receiver Operating Characteristic (ROC) Curves of HC vs OCD Classifiers Based on Different Combinations of Brain Metrics
eReferences.

Supplement 2.
Data Sharing Statement
