# Supplementary material for: Pathological Networking of Gray Matter Dendritic Density With Classic Brain Morphometries in OCD
Source: JAMA Netw Open. 2023 Nov 13;6(11):e2343208. doi: 10.1001/jamanetworkopen.2023.43208 (PMC10644219; doi:10.1001/jamanetworkopen.2023.43208)
Supplement: Supplement 1. — eMethods 1. Eligibility and Exclusion Criteria eMethods 2. Magnetic Resonance Image Acquisition eMethods 3. Processing of Multishell Diffusion-Weighted MR Images eMethods 4. Processing of Single-Shell Diffusion-Weighted MR Images eMethods 5. Processing of T1 Images eMethods 6. Whole Brain Analyses of Morphological Metrics eMethods 7. Post Hoc Anatomical Connectivity Analyses eMethods 8. Post Hoc Construction of Networks of Brain Metrics eMethods 9. Post Hoc Analyses of Networks of Brain Metrics eMethods 10. Post Hoc Correlational Analyses With Symptoms eMethods 11. Post Hoc Machine Learning-Based OCD vs HC Classification eAppendix 1. Demographic Characteristics eAppendix 2. OCD-Associated Alterations of Gray Matter Microstructure eAppendix 3. OCD-Associated Alterations of Gray Matter Morphology eAppendix 4. OCD-Associated Alterations of White Matter Morphology and Microstructure eAppendix 5. Emergence of a Pathological Brain Network Among Patients With OCD eAppendix 6. Correlations Between Nodes of OCD Pathological Brain Network and Clinical Symptoms eAppendix 7. Performance of Pathological Brain Network in Identifying OCD Patients eAppendix 8. Supplemental Discussion eTable 1. Gray Matter Volume Alterations in Patients With OCD eTable 2. Gray Matter Surface Morphological Alterations in Patients With OCD eTable 3. Performance Comparison of Brain Metrics for OCD Patient Identification eTable 4. Performance Comparison of Brain Metric Combinations for OCD Patient Identification eFigure 1. The Complete Workflow for Analyzing Neuroimaging Data eFigure 2. Results of the NODDI-GBSS Analysis eFigure 3. Results of the Voxel-Based Morphometry eFigure 4. Results of the Surface-Based Morphometries eFigure 5. Results of the White Matter TBSS Analysis eFigure 6. Correlations Between Clinical Symptoms and Nodes of the Pathological Brain Network in OCD Patients eFigure 7. Receiver Operating Characteristic (ROC) Curves of HC vs OCD Classifiers Based on Different Combinations of Brai [file jamanetwopen-e2343208-s001.pdf]

Supplemental Online Content

Zhang X, Zhou J, Chen Y, et al. Pathological networking of gray matter dendritic density with classic brain morphometries in OCD. *JAMA Netw Open*. 2023;6(11):e2343208. doi:10.1001/jamanetworkopen.2023.43208

eMethods 1. Eligibility and Exclusion Criteria
eMethods 2. Magnetic Resonance Image Acquisition
eMethods 3. Processing of Multishell Diffusion-Weighted MR Images
eMethods 4. Processing of Single-Shell Diffusion-Weighted MR Images
eMethods 5. Processing of T1 Images
eMethods 6. Whole Brain Analyses of Morphological Metrics
eMethods 7. Post Hoc Anatomical Connectivity Analyses
eMethods 8. Post Hoc Construction of Networks of Brain Metrics
eMethods 9. Post Hoc Analyses of Networks of Brain Metrics
eMethods 10. Post Hoc Correlational Analyses With Symptoms
eMethods 11. Post Hoc Machine Learning-Based OCD vs HC Classification
eAppendix 1. Demographic Characteristics
eAppendix 2. OCD-Associated Alterations of Gray Matter Microstructure
eAppendix 3. OCD-Associated Alterations of Gray Matter Morphology
eAppendix 4. OCD-Associated Alterations of White Matter Morphology and Microstructure
eAppendix 5. Emergence of a Pathological Brain Network Among Patients With OCD
eAppendix 6. Correlations Between Nodes of OCD Pathological Brain Network and Clinical Symptoms
eAppendix 7. Performance of Pathological Brain Network in Identifying OCD Patients
eAppendix 8. Supplemental Discussion
eTable 1. Gray Matter Volume Alterations in Patients With OCD
eTable 2. Gray Matter Surface Morphological Alterations in Patients With OCD
eTable 3. Performance Comparison of Brain Metrics for OCD Patient Identification
eTable 4. Performance Comparison of Brain Metric Combinations for OCD Patient Identification
eFigure 1. The Complete Workflow for Analyzing Neuroimaging Data
eFigure 2. Results of the NODDI-GBSS Analysis
eFigure 3. Results of the Voxel-Based Morphometry
eFigure 4. Results of the Surface-Based Morphometries

© 2023 Zhang X et al. *JAMA Network Open.*

eFigure 5. Results of the White Matter TBSS Analysis
eFigure 6. Correlations Between Clinical Symptoms and Nodes of the Pathological Brain Network in OCD Patients
eFigure 7. Receiver Operating Characteristic (ROC) Curves of HC vs OCD Classifiers Based on Different Combinations of Brain Metrics
eReferences.

This supplemental material has been provided by the authors to give readers additional information about their work.

© 2023 Zhang X et al. *JAMA Network Open*.

eMethods

1. Eligibility and Exclusion Criteria

Eligibility criteria required participants to be aged between 18-54 years, right-handed, have at least a junior high school education level, have sufficient audiovisual abilities for exams, and have voluntarily participated in the study. Exclusion criteria were in place to exclude participants with current serious physical or neurological disorders, substance abuse, pregnancy or lactation, or contraindications for MRI scanning, such as metal implants or claustrophobia. Besides, patients with a DSM-IV Axis I diagnosis other than OCD, and those who reported taking psychiatric medication within the past 8 weeks were excluded. Additional exclusion criteria for healthy volunteers included reporting past use of psychiatric medication, a history of psychiatric disorders in siblings or within two generations on either parental side, or a high risk of suicide.

2. Magnetic Resonance Image Acquisition

All MR images were acquired utilizing a 3.0T magnetic resonance imaging system (Verio, Siemens) at the radiology department of Shanghai Mental Health Center[1]. T1-weighted structural images were obtained using a three-dimensional magnetization-prepared rapid acquisition gradient-echo (MPRAGE) sequence, with the following acquisition parameters: an echo time (TE) of 2.96 ms, a repetition time (TR) of 2300 ms, an inversion time (TI) of 900 ms, a flip angle of 9°, a resolution of 1×1×1 mm^3, an acquisition matrix of 256×240 voxels, and a total of 192 slices. The High Angular Resolution Diffusion Imaging (HARDI) data were acquired utilizing an Echo Planar Imaging (EPI) sequence, with two different b values (64 directions each) of 1000 and 2000 s/mm^2, and a b_0 image at the beginning. The scanning parameters were as follows: an echo time of 110 ms, a repetition time of 10100 ms, a resolution of 2×2×3.3 mm^3, an acquisition matrix of 110×110 voxels, and a total of 42 slices.

eFigure 1 illustrates the complete workflow of analyzing the neuroimaging data.

3. Processing of Multi-shell Diffusion-Weighted MR Images

The multi-shell diffusion-weighted images were utilized to assess the morphology of neurites. We first utilized the *eddy_correct* function of FSL (version 5.0.10) to correct for motion-related misalignments and eddy current distortions in the multi-shell diffusion-weighted images. A brain mask was subsequently created from the b0 images using the Brain Extraction Tool. For the quantification of neurite morphology, we employed the NODDI technique[2, 3], a biophysical diffusion modeling approach that differentiates the intracellular (neuritic), extracellular, and cerebrospinal fluid (CSF) compartments of neurite microstructure from multi-shell diffusion-weighted MR images. The NDI map was generated to calculate the proportion of tissue water restricted within neurites (axons and dendrites) in the non-CSF compartment, and the ODI map was derived to characterize the spatial configuration of neurites, ranging from 0 (no neuritic dispersion) to 1 (full dispersion). These maps were employed in further analyses to investigate alterations in neurite morphology associated with OCD. While the map of fraction of CSF (fCSF), quantifying the volume fraction of Gaussian isotropic diffusion (free fluid) within each voxel, was also generated, it was not subjected to further analysis.

We employed the NODDI GM-based spatial statistics (NODDI-GBSS) method[4], which has previously been used to examine microstructural changes in schizophrenia and bipolar disorder[3], to skeletonize the NODDI-derived parameter maps. The detailed procedure of NODDI-GBSS has been

© 2023 Zhang X et al. *JAMA Network Open.*

described in prior literature[3, 4], and the GBSS pipeline scripts are accessible to the public (https://github.com/arash-n/GBSS). Here, we only provide a concise overview of the NODDI-GBSS. Firstly, GM fraction maps were generated in the native diffusion space by subtracting the CSF and WM fraction from 1 in each voxel. Partial volume estimation maps were multiplied by their corresponding contrast and summed together to increase tissue contrasts and improve between-subject registration. The resulting images were utilized to develop a study-specific template, and GM fraction, ODI, and NDI images were transformed to the template space using warp fields. GBSS adopted the tract-based spatial statistics (TBSS) algorithm to align GM voxels between subjects. The average GM fraction map was skeletonized, and diffusion metrics and GM fraction were projected onto the skeleton for each subject. Voxels with satisfactory GM fraction (>0.65) were retained on the skeleton, and those with non-satisfactory GM fraction were filled with the average of the surrounding satisfactory voxels.

4. Processing of Single-shell Diffusion-Weighted MR Images

In addition, conventional diffusion tensor imaging (DTI) measures[5], including fractional anisotropy (FA), mean diffusivity (MD), axial diffusivity (AD), and radial diffusivity (RD), were also utilized to quantify the microstructural features of the white matter. These metric maps were prepared by PhiPipe[6], which uses the Tract-Based Spatial Statistics (TBSS)[7, 8] functions available in FSL. To obtain a mean FA skeleton, each participant's FA map was aligned into a common space using nonlinear registration and then averaged to create a mean FA map, which was subsequently thinned to represent the center of all tracts common to this group of participants. The individual FA data and other metrics, including MD, AD, RD, NDI, and ODI, were then projected onto the skeleton.

5. Processing of T1 Images

The T1-weighted brain images were subjected to the conventional voxel-based morphometry (VBM)[9] and surface-based morphometry (SBM) procedures utilizing the Computational Anatomy Toolbox (CAT12) and Statistical Parametric Mapping (SPM12) toolboxes, both implemented in Matlab (R2019b).

Firstly, the images underwent routine segmentation into gray matter (GM), white matter (WM), and cerebrospinal fluid (CSF). The GM images were subsequently normalized to Montreal Neurological Institute (MNI) space using high-dimensional Diffeomorphic Anatomical Registration Through Exponentiated Lie Algebra (DARTEL), yielding maps with a spatial resolution of $1.5\times1.5\times1.5$ mm^3. A modulation process was performed on the GM probability maps to adjust for the impact of spatial normalization on volumetric data. Subsequently, the GM images underwent smoothing utilizing an 8-mm full-width at half-maximum (FWHM) isotropic Gaussian kernel. The preprocessed GM probability maps were prepared for voxel-wise whole-brain analyses.

In addition to the GM probability maps, surface-based morphological measures were obtained using the automated surface-preprocessing algorithms incorporated in the CAT12 toolbox. This process involved a projection-based thickness estimation that facilitated the computation of cortical thickness and reconstruction of the central surface of both hemispheres concurrently[10]. A gyrification index was extracted based on absolute mean curvature[11]. The cortical complexity[12] and sulcal depth[13] measures were also derived, defined as the fractal dimension and the Euclidean distance between the central surface and its convex hull, respectively. The surface meshes were then re-parameterized into a common coordinate system using spherical maps to facilitate inter-subject comparisons. Finally, all surface measures were resampled, merged between both hemispheres, and smoothed with a Gaussian

© 2023 Zhang X et al. *JAMA Network Open.*

kernel of 15 mm (FWHM) for cortical thickness and 20 mm (FWHM) for other parameters. The extracted surface measures were prepared for vertex-wise whole-brain analyses.

The CAT12 toolbox also conducted a quality assessment of the T1 brain images and provided an estimate of the total intracranial volume (TIV).

6. Whole Brain Analyses of Morphological Metrics

Voxel-wise permutation analysis was conducted on GBSS-derived skeletonized NODDI parameter maps across participants using the *randomise* function of FSL to test the differences between the two diagnostic groups while controlling for age, sex, and education. The threshold-free cluster enhancement (TFCE) method[14] was utilized for each permutation analysis to identify brain-wide statistical significance corrected for multi-comparisons without an arbitrary cluster threshold.

Differences in the TBSS-derived white matter morphological measures between individuals with OCD and healthy controls were tested using voxel-wise comparisons, utilizing the same statistical model and multi-comparison correction as previously stated for the GBSS-derived skeletonized NODDI parameter maps.

Apart from neurite morphology, we also assessed the differences between the two groups in conventional metrics of gray matter morphology using two different types of whole brain analyses: a voxel-wise analysis of gray matter volume and vertex-wise analyses of various surface-based morphological metrics, including cortical thickness, gyrification, complexity, and sulcal depth. We used parametric two-sample t-tests in SPM12, with age, sex, and education included as covariates to control for potential confounding factors. In the voxel-wise analysis of gray matter volume, total intracranial volume (TIV) was also controlled, and a probability threshold of >0.2 was set as the definition of gray matter. We then applied a voxel-level threshold of $p < 0.001$ (also known as cluster-producing threshold) and a cluster-level threshold of $p < 0.05$ corrected for the familywise error (FWE) to identify significant clusters.

If whole brain analyses of morphological metrics revealed significant alterations in patients with OCD, the clusters where significant alterations occurred were defined as ROIs, and their values were averaged for data visualization and further analyses. An ANOVA with diagnostic group, sex, age, education, and TIV (for gray matter volume ROIs) as between-subject factors was performed for each ROI in SPSS (version 26).

7. Post-hoc Anatomical Connectivity Analyses

Given that morphological alterations in gray matter dendrites may affect the anatomical connectivity of brain regions exhibiting such alterations, we investigated whether the degree of centrality of these regions was also impaired. Degree centrality represents the overall anatomical connectivity of a given region to all other regions. In addition, extensive alterations in white matter microstructure may affect interregional anatomical connectivity widely. Hence, we also investigated whether the global anatomical connectivity strength was impaired. Global anatomical connectivity strength represents the overall anatomical connectivity between all possible pairs of cortical regions.

To achieve these goals, the probabilistic fiber tracking algorithm[15] was executed within PhiPipe[6], which called relevant functions from FSL with the default parameters. The probabilistic fiber tracking was based on the single-shell diffusion-weighted images (b = 1000 s/mm^2) and the b0 image in the native space of each participant. The Desikan-Killiany Atlas[16] was used to define 68 cortical regions as the seeds for fiber tracking. From each seed, the probability of a connection to adjacent voxels was

© 2023 Zhang X et al. *JAMA Network Open.*

estimated, and the algorithm propagated along the estimated fiber pathway, producing a probability estimate of anatomical connections to other brain regions through white matter tracts. Ultimately, a 68×68 matrix of anatomical connectivity probability was generated for each participant.

Subsequently, we computed the degree centrality of the brain regions corresponding to gray matter ROIs or the global strength of anatomical connectivity, and carried out ANOVA with diagnostic group, sex, age, and education as between-subject factors to investigate the potential impairments among individuals with OCD. In the event of a confirmed impairment, we delved further to examine whether the impairment in anatomical connectivity was mediated by brain morphological metrics, using mediation analyses[17]. Specifically, the diagnostic group served as the independent variable (X), degree centrality as the outcome variable (Y), and morphological metrics as mediating variables (M). We utilized Model 4 and 6 of the PROCESS toolbox[18] (release 2.11) implanted in SPSS for mediation analyses, employing 10000 bootstrap samples and sex, age, and education as covariates for both M and Y.

8. Post-hoc Construction of Networks of Brain Metrics

To investigate the interplay between different brain morphological alterations related to OCD, we examined whether morphological metrics that significantly changed among OCD patients are differentially interlinked between the patient and the control groups, through a network analysis[19]. The Graphical Gaussian Model (GGM) was used to estimate the network, which may lead to some false-positive edges. To overcome this issue, we used the graphical lasso (glasso) algorithm to shrink the edges, leading to a sparse network that explains the covariance among nodes with as few edges as possible. We used the R package *bootnet* to estimate the GGMs and automatically implemented the glasso regularization with extended Bayesian Information Criterion (EBIC) model selection to select the best model. The starting value of the hyperparameter γ was set to 0.5 as default. The magnitude of the association between the nodes was represented by the thickness of the edges for network visualization.

9. Post-hoc Analyses of Networks of Brain Metrics

Here, we constructed two separate networks of brain morphological metrics, one for healthy controls and one for individuals with OCD, to examine whether these networks exhibited dissimilar structures. We employed two approaches. Firstly, we compared the stability of the two networks by estimating the stability of edge weights by computing the 95% confidence intervals[20]. We used a case-dropping subset bootstrapping procedure for strength centrality and calculated the correlation stability coefficient (CS-coefficient). A CS-coefficient above 0.25 indicates acceptable stability, but we prefer it to be above 0.5[20]. Our hypothesis posited that the network stability would be high for OCD patients and low for healthy controls. Secondly, we assessed global network strength and network structure to identify changes in highly connected nodes that would suggest significant structural differences. We employed the Network Comparison Test (NCT) to examine changes in global parameters[20]. The NCT involved conducting permutation tests in which group members were randomly reassigned to new subsamples with the same original sample sizes, and comparing the network structures of the two groups 2000 times to evaluate the null distributions.

10. Post-hoc Correlational Analyses with Symptoms

© 2023 Zhang X et al. *JAMA Network Open.*

In this study, we employed Spearman's rank correlation coefficient rho to assess the correlations between brain morphological metrics and symptoms in OCD patients, given the non-normal distribution of some data. The symptoms were assessed using the seven scores from the scales and inventories detailed in section 2.1, with occasional missing values among participants. A statistical significance level of $p < 0.05$ was adopted; both unadjusted and false discovery rate (FDR) corrected p-values were reported for the post-hoc correlational analyses of each brain metric with the multiple symptom scores. We conducted one-sided tests based on the direction of association inferred from the comparison with healthy controls. The expected outcome was that greater abnormalities in symptoms would positively correlate with greater changes in brain metrics. However, two-sided tests were conducted in situations where the direction of the correlation contradicted what was inferred from the comparison with healthy controls.

To investigate the possible relationship between the comprehensive information of all brain metrics representing morphological alterations related to OCD and the overall severity of the symptoms, we applied unsupervised hierarchical clustering[21] to segregate the patients with OCD into distinct subgroups based entirely on their brain data. Subsequently, we investigated whether any discrepancies in the Y-BOCS total scores could be observed among these subgroups. We performed unsupervised hierarchical clustering using SPSS with median linkage and Euclidean distance. Median linkage was chosen for its resistance to potential outliers. The number of subgroups was determined through a visual inspection of the dendrogram.

11. Post-hoc Machine Learning-based OCD vs. HC Classification

We investigated the performance of various combinations of brain metrics in discriminating OCD patients from healthy individuals, addressing the practical question of whether multiple modalities of brain imaging could facilitate the development of neuroimaging-based biomarkers. Firstly, we assessed the performance of individual brain metrics as input variables, followed by evaluating four combinations of brain metrics obtained from the four metric deriving procedures (NODDI+GBSS, DTI+TBSS, VBM, and SBM), two combinations of brain metrics from the two image modalities (diffusion and T1), and all previously identified brain metrics. Two types of classifiers, logistic regression and support vector machine, were employed for the classification. Classification performance was evaluated by the area-under-the-curve (AUC) value, the accuracy, the sensitivity, and the specificity for each run of classification using a leave-one-out cross-validation. Here, the classifier learner implanted in Matlab was utilized for training classifiers.

© 2023 Zhang X et al. *JAMA Network Open*.

eAppendix

1. Demographic Characteristics

As shown in Table, the two diagnostic groups were demographically matched. The percentage of female participants was 46% for HC and 43% for OCD, and showed no significant differences between the two groups (χ^2=0.300; p=0.584). The median (interquartile range, IQR) age was 26 (23-31) years for HC and 26 (24-31) years for OCD. The distribution of age was not significantly different between the two groups (Kolmogorov–Smirnov statistic=0.680; p=0.744). The median (IQR) education was 16 (15-17) years for HC and 16 (15-16) years for OCD. The distribution of education was not significantly different between the two groups (Kolmogorov–Smirnov statistic=1.293; p=0.071).

In addition, OCD patients exhibited more severe depression and anxiety compared with healthy participants. Specifically, OCD patients manifested higher HDRS scores ($F_{1, 199}$ = 90.395, p < 0.001, η^2 = 0.312), higher HARS scores ($F_{1, 200}$ = 79.721, p < 0.001, η^2 = 0.285), higher state anxiety score ($F_{1, 188}$ = 88.167, p < 0.001, η^2 = 0.319), and higher trait anxiety ($F_{1, 190}$ = 193.652, p < 0.001, η^2 = 0.505), after adjusting for sex, age, and education.

2. OCD-related Alterations of Gray Matter Microstructure

Utilizing gray matter-based spatial statistics (GBSS) analysis throughout the brain, we discovered a significant deficit in the neurite density index (NDI) among patients diagnosed with OCD in comparison to healthy participants, specifically in the superior section of the right lateral occipital cortex (peak T = 3.821; peak p_{tfce} = 0.026) and the right angular gyrus (peak T = 3.446; peak p_{tfce} = 0.029), extending to the posterior division of the right supramarginal gyrus (peak T = 2.292; peak p_{tfce} = 0.036), as demonstrated in Figure 1, while controlling for age, sex, and education. Following this, we defined voxels showing significantly lower GM-NDI in the GBSS as an ROI and calculated the average NDI values within it for subsequent analyses. As demonstrated in eFigure 2A, the averaged NDI within the ROI was significantly lower among OCD patients than healthy participants ($F_{1, 211}$ = 74.198, p < 0.001, η^2 = 0.260), after controlling for age, sex, and education. We did not observe any significant differences between the two groups in the orientation dispersion index (ODI).

Then, we conducted post-hoc investigations into whether changes in the degree centrality of the right lateral occipital and inferior parietal cortices were present in patients with OCD, and whether these changes were mediated by alterations in local NDI. The degree centrality was quantified by the summed probability of anatomical connectivity from the two cortical regions to others. We found that patients with OCD showed a deficit in degree centrality of the right lateral occipital and inferior parietal cortices ($F_{1, 211}$ = 4.245, p = 0.041, η^2 = 0.020), after adjusting for age, sex, and education. This indicates a significant impairment in the anatomical connectivity linking these two regions to others. A further mediation analysis revealed that this anatomical connectivity alteration was completely mediated by the local NDI (indirect effect = –0.0240, 95% CI = [–0.0508, –0.0019]; direct effect = –0.0058, 95% CI = [–0.0384, 0.0268]; total effect = –0.0298, 95% CI = [–0.0583, –0.0013]), as shown in eFigure 2B. Together, these findings suggest that the deficient dendritic density at the right occipitoparietal regions probably in turn hinder their anatomical connections to other brain regions.

3. OCD-related Alterations of Gray Matter Morphology

Initially, classic voxel-based morphometry (VBM) was implemented across the whole brain to determine areas where gray matter volume was abnormally altered in patients with OCD. Following

© 2023 Zhang X et al. *JAMA Network Open*.

control of age, sex, and education, there was no significant difference in total intracranial volume (TIV) between healthy participants and OCD patients. Therefore, we proceeded to investigate the differences between the two groups in the relative volume of local gray matter. In comparison to healthy individuals, OCD patients displayed significantly deficient gray matter volume in the left medial parietal structures (i.e., the precuneus and the posterior cingulate gyrus; peak T = 4.62, $p_{FWE\text{-}cluster}$ = 0.001) and right medial frontal structures (i.e., the medial orbital gyrus and the gyrus rectus; peak T = 4.49, $p_{FWE\text{-}cluster}$ = 0.034), as illustrated in Figure 2A, 2B, and eTable 1, with TIV, age, sex, and education controlled. Subsequently, two ROIs were defined from the two clusters of voxels exhibiting significantly diminished gray matter in the VBM. As demonstrated in eFigure 3A and 3B respectively, the averaged gray matter volume within the left medial parietal ROI ($F_{1, 211}$ = 22.919, $p < 0.001$, η^2 = 0.098) and the right medial frontal ROI ($F_{1, 211}$ = 20.234, $p < 0.001$, η^2 = 0.088) was significantly lower in patients with OCD than in healthy individuals, with TIV, age, sex, and education controlled.

We subsequently employed a series of surface-based morphometry (SBM) analyses across the cerebral cortex to identify regions exhibiting abnormal cortical thickness, local gyrification, complexity (i.e., fractal dimension), or sulcal depth in patients with OCD compared to healthy participants. Controlling for age, sex, and education, patients with OCD demonstrated significantly deficient cortical thickness in the left fusiform gyrus (peak T = 4.35, $p_{FWE\text{-}cluster}$ = 0.039) and disrupted local gyrification in the right lateral frontal structures (i.e., the right superior and middle frontal gyri; peak T = 3.73, $p_{FWE\text{-}cluster}$ = 0.008) and the left middle cingulate gyrus (peak T = 3.61, $p_{FWE\text{-}cluster}$ = 0.016), as presented in eFigure 4A, 4B, 4C, and eTable 2. However, we did not observe any significant differences between the two groups in the fractal dimension or sulcal depth.

To further analyze the regions showing significantly aberrant gray matter morphology, we defined three clusters of vertices in the SBMs as three ROIs and averaged the morphological values within each of them. As shown in eFigure 4A, 4B, and 4C, patients with OCD had significantly deficient cortical thickness in the left fusiform ROI ($F_{1, 211}$ = 19.594, $p < 0.001$, η^2 = 0.085) and disrupted local gyrification in the right lateral frontal ROI ($F_{1, 211}$ = 18.734, $p < 0.001$, η^2 = 0.082) and the left middle cingulate ROI ($F_{1, 211}$ = 15.088, $p < 0.001$, η^2 = 0.067), after controlling for age, sex, and education.

The degree centrality of the left precuneus and posterior cingulate cortices (with alterations in gray matter volume), that of the right orbitofrontal cortices (with alterations in gray matter volume), that of the left fusiform cortex (with alterations in thickness), that of the right caudal middle frontal and superior frontal cortices (with alterations in gyrification), and that of the left caudal anterior cingulate cortex (with alterations in gyrification) were not significantly altered among patients with OCD, and thus further mediation analyses were not performed. These results suggest that the morphological alterations indexed by conventional metrics do not necessarily indicate anatomical connectivity deficits.

4. OCD-related Alterations of White Matter Morphology and Microstructure

Through the use of TBSS across the brain, we have identified an extensive area of significantly greater white matter AD among patients with OCD compared to healthy participants, which is present in both hemispheres (peak T = 4.852; peak p_{tfce} = 0.006), as presented in eFigure 5A. Additionally, we found abnormally greater white matter MD in less extensive regions lateralized to the right hemisphere (peak T = 4.797; peak p_{tfce} = 0.029), as demonstrated in eFigure 5C. After controlling for age, sex, and education, we defined the voxels displaying significantly greater AD and MD in TBSS as two ROIs and calculated the averaged diffusivity values within each of them for further analyses. As shown in

eFigure 5B and 5D, patients with OCD exhibited a significantly higher average AD ($F_{1, 211}$ = 47.556, p < 0.001, η^2 = 0.184) and a higher average MD ($F_{1, 211}$ = 47.221, p < 0.001, η^2 = 0.183) in the ROIs compared to healthy participants, after controlling for age, sex, and education. However, we did not observe any significant differences between the two groups in white matter FA, RD, NDI, or ODI.

Furthermore, we observed a significant impairment in the global strength of anatomical connectivity between all possible pairs of cortical regions among patients with OCD ($F_{1, 211}$ = 5.235, p = 0.023, η^2 = 0.024), after controlling for age, sex, and education. However, the global strength of anatomical connectivity between the cortical regions where we previously found morphological alterations in this study was not significantly altered among patients with OCD ($F_{1, 211}$ = 2.411, p = 0.122, η^2 = 0.011), after controlling for age, sex, and education. Instead, the overall strength of anatomical connectivity between the cortical regions where we previously observed morphological alterations in this study and the remaining cortical regions was significantly impaired in patients with OCD ($F_{1, 211}$ = 4.431, p = 0.036, η^2 = 0.021), after controlling for age, sex, and education. Further mediation analyses revealed that the AD and MD in the extensive regions of corresponding ROIs partially mediated, in a serial manner, the impairment in the global strength of anatomical connectivity between all possible pairs of cortical regions (indirect effect = –0.2045, 95% CI = [–0.4911, –0.0655]; direct effect = –0.8590, 95% CI = [–1.3663, –0.3518]; total effect = –0.5556, 95% CI = [–1.0342, –0.0769]), as shown in eFigure 5E, as well as the impairment in the overall strength of anatomical connectivity linking the cortical regions where we previously observed morphological alterations in this study to the remaining cortical regions (indirect effect = –0.0595, 95% CI = [–0.1341, –0.0045]; direct effect = –0.2249, 95% CI = [–0.3872, –0.0627]; total effect = –0.1596, 95% CI = [–0.3090, –0.0101]).

5. Emergence of a Pathological Brain Network among Patients with OCD

Subsequently, we proceeded to examine the relationships between these metrics by conducting network analyses based on the covarying relationship between the previously identified brain morphological metrics in both healthy individuals and OCD patients. A distinct difference was observed in the inter-metric connections between healthy participants and patients with OCD, as shown in Figure 3. In healthy participants, only a positive correlation was observed between the GMV at the right medial frontal regions and the GMV at the left medial parietal regions (edge weight w = 0.253), while the other brain metrics were not associated with each other. On the other hand, among patients with OCD, all the eight brain metrics were correlated with at least one other brain metric. Specifically, the GMV at the right medial frontal regions was positively correlated with the GMV at the left medial parietal regions (w = 0.443), the cortical thickness at the left fusiform gyrus (w = 0.112), and the white matter AD (w = 0.082). The white matter AD and the white matter MD were highly correlated with each other (w = 0.572). The GM-NDI at right lateral occipitoparietal regions was negatively correlated with the white matter MD (w = –0.022). The gyrification at the right lateral frontal regions was negatively correlated with the cortical thickness at the left fusiform gyrus (w = –0.044). Also, the gyrification at the left middle cingulate gyrus was negatively correlated with the GMV at the left medial parietal regions (w = –0.080) and the white matter AD (w = –0.121).

We utilized a network stability analysis to examine the stability of the brain metric network in OCD patients compared to healthy individuals. The results revealed a substantial difference in the CS-coefficient of the network of brain metrics between the two groups, with a coefficient of 0.593 for patients with OCD indicating a stable network, while the coefficient of 0.046 for healthy participants

© 2023 Zhang X et al. *JAMA Network Open*.

indicated an unstable network. Moreover, a comparison analysis of network structure revealed a higher global strength of the network of brain metrics in patients with OCD than in healthy individuals (sum of all edge weights: HC = 0.253, OCD = 0.941, p = 0.046), and a significant structural difference between the two networks (maximum edge difference = 0.572, p < 0.001). Four pairs of nodes showed significantly altered edge weight between the two groups, namely, the gyrification at the right lateral frontal regions and the cortical thickness at the left fusiform gyrus ($|\Delta w|$ = 0.044, p = 0.043), the gyrification at the left middle cingulate gyrus and the GMV at the left medial parietal regions ($|\Delta w|$ = 0.080, p = 0.005), the GMV at the right medial frontal regions and the white matter AD ($|\Delta w|$ = 0.082, p = 0.015), and the white matter AD and the white matter MD ($|\Delta w|$ = 0.572, p < 0.001). These findings suggest that the dissociated brain metrics in healthy participants were pathologically connected among patients with OCD, indicating the emergence of a pathological brain network unique to patients with OCD, but not present in healthy individuals.

To investigate whether the observed group differences in inter-metric correlations stemmed from differences in individual brain metrics between groups, we employed a linear regression model to remove the main effect of diagnostic group from each brain metric. Subsequently, we subjected the residual values, instead of the raw values, to the network analysis. This modified approach yielded results that closely mirrored the initial findings, with one notable exception: an additional pair of nodes, the gyrification at the left middle cingulate gyrus and the white matter AD, exhibited a significantly altered edge weight between the two groups ($|\Delta w|$ = 0.121, p = 0.013).

6. Correlations between Nodes of OCD Pathological Brain Network and Clinical Symptoms

We investigated the correlations between clinical symptoms and nodes of the pathological brain network in OCD patients. The brain alterations of larger extent tended to be correlated with more severe symptoms, indicating a relationship between brain metrics and symptomatology. Specifically, the NDI at the right lateral occipitoparietal regions tended to be negatively correlated with state anxiety (rho = –0.171, df = 93, p = 0.049, p_{FDR} = 0.345; eFigure 6A), while the GMV at the left medial parietal regions was negatively correlated with the Y-BOCS compulsion score (rho = –0.245, df = 106, p = 0.005, p_{FDR} = 0.037; eFigure 6B). Moreover, the GMV at the right medial frontal regions was negatively correlated with depression (rho = –0.239, df = 105, p = 0.006, p_{FDR} = 0.045; eFigure 6C) and perhaps also with trait anxiety (rho = –0.178, df = 95, p = 0.041, p_{FDR} = 0.142; eFigure 6D), and the gyrification at the left middle cingulate gyrus tended to be negatively correlated with depression (rho = –0.167, df = 105, p = 0.043, p_{FDR} = 0.301; eFigure 6E). White matter MD tended to be negatively correlated with trait anxiety (rho = –0.242, df = 95, p = 0.017, p_{FDR} = 0.119, two-sided; eFigure 6F).

Moreover, the study revealed that all eight nodes of the pathological brain network, together, could effectively distinguish patients with higher overall severity from those with less severe overall symptoms, as measured by the Y-BOCS total score. Specifically, unsupervised hierarchical clustering allowed for the classification of OCD patients into two subgroups based on the eight brain metrics (eFigure 6G). These subgroups did not significantly differ in age, education, or any clinical symptom except for the Y-BOCS total score (subgroup 1 < subgroup 2, t = –2.192, p = 0.031; eFigure 6H), suggesting that the overall severity of OCD can be effectively captured by the combination of brain metrics. As there was a significant difference in the sex distribution between the OCD patient groups (χ^2 = 8.928, p = 0.003), we created a subsample (N = 88) of OCD patients that were matched in terms of age, sex, and education. After this resampling, the two subgroups still differed significantly only in the Y-BOCS total score (subgroup 1 < subgroup 2, t = –2.057, p = 0.043; eFigure 6I). These findings

© 2023 Zhang X et al. *JAMA Network Open.*

suggest that the different nodes of the pathological brain network in OCD patients are associated with distinct clinical symptoms and provide relevant brain information for the assessment of the overall severity of OCD.

7. Performance of Pathological Brain Network in Identifying OCD Patients

To evaluate the effectiveness of the pathological brain network for distinguishing individuals with OCD from healthy participants, we conducted two comparisons. Firstly, we examined the performance of every node of the pathological brain network for this purpose. Our analysis revealed that the NDI located at the right occipitoparietal regions outperformed other brain metrics in terms of classification accuracy, area-under-the-curve (AUC) values, and sensitivity, and was the second best in terms of specificity, as shown in eTable 3. Next, we compared the performances of node clusters derived from different feature extraction procedures. The results were listed in eTable 4. Specifically, we evaluated nodes derived from NODDI+GBSS and DTI+TBSS based on diffusion images, as well as nodes derived from VBM and SBM based on T1 images. The receiver operating characteristic curves of all the mentioned classifiers are given in eFigure 7.

Our findings indicated that the node derived from NODDI+GBSS outperformed the nodes derived from DTI+TBSS, and combining them led to improved classification performance. Similarly, the nodes derived from SBM outperformed the nodes derived from VBM. We also compared the performance of node clusters derived from brain images of different modalities and found that combining the node clusters from diffusion images resulted in superior classification performance than combining those from T1 images. Importantly, the performance of the node derived from NODDI+GBSS exceeded that of all nodes derived from T1 images combined. The highest classification performance was achieved by using the entire network, with an accuracy ≥ 81.48%, an AUC ≥ 0.905, a sensitivity ≥ 0.824, and a specificity ≥ 0.805. These results highlight the potential of the metric derived from NODDI+GBSS, which reflects the gray matter neurite morphology, in the development of biomarkers for OCD based on the neuro-phenomenology of OCD, as well as the overall classification performance of the brain metrics constructing the pathological brain network for OCD.

© 2023 Zhang X et al. *JAMA Network Open*.

eAppendix 8. Supplemental Discussion

Resistance of Post-hoc Network Analysis to Selective Inference Issues

In this study, the data chosen for the network analysis initially underwent comprehensive whole-brain searches aimed at identifying group main effects between the patient and control groups. The subsequent network analysis served as a logical, post-hoc exploration of the relationships among the identified brain metrics exhibiting alterations associated with the disorder. These relationships offer potential insights into latent pathological or etiological factors. However, it is essential to acknowledge that the selection of input data could raise concerns about susceptibility to the selective inference problem[22]. This problem revolves around the possibility of inflating statistical significance due to data selection.

It is worth noting that that reusing selected data does not inherently lead to issues related to the selective inference problem, as long as the subsequent analysis focuses on "effects shown to be statistically independent of the selection criterion"[23]. In this study, the network analysis specifically assessed differences in inter-metric correlations between the two groups. This is independent of the group main effect on each individual brain metric, which was the basis for selecting the data for network analysis initially.

Presumably, significant differences across groups in any given brain metric do not necessarily imply, cause, or result from significant differences across groups in the correlation between brain metrics. Furthermore, it is not universally assumed or empirically confirmed that correlations between altered brain metrics related to a disorder will consistently demonstrate significant differences between healthy individuals and patients. For instance, in the context of OCD, the brain metrics identified as altered in comparison to healthy controls by the ENIGMA Consortium[24, 25] are not the same metrics whose correlations with other brain metrics (indexed by centrality) were found to be altered compared to healthy controls, as also reported by the ENIGMA Consortium[26], demonstrating that brain metrics with structural alterations may not necessarily exhibit correlated alterations with other brain metrics. These findings strongly suggest that the effects addressed in our network analysis are independent of the criteria used for selecting input data.

In addition, as a precautionary step, we regressed out the main effect of diagnostic group for each brain metric using a linear regression model, and then subjected the residual values, rather than the raw values, to the network analysis. The results of this approach closely mirrored the initial findings, further underscoring that the main effect of diagnostic group on the brain metrics is minimally related to the correlations among them.

Taking into account all of the aforementioned considerations, we are confident that our network analysis is reasonably immune to the selective inference problem.

© 2023 Zhang X et al. *JAMA Network Open.*

eTables

eTable 1. Gray Matter Volume Alterations in Patients with OCD

Cluster	$p_{FWE\text{-}cluster}$	Number of voxels	Local maxima				Labels
			T	x (mm)	y (mm)	z (mm)	
1	0.001	1916	4.62	–3	–52	40	L Precu
			4.32	–4	–44	33	L Post Cing Gyr
			3.86	–9	–56	27	L Precu
2	0.034	868	4.49	6	52	–22	R Gyr Rectus
			4.11	9	45	–20	R Med Orb Gyr
			3.80	10	33	–20	R Med Orb Gyr

Abbreviations: L Post Cing Gyr, left posterior cingulate gyrus; L Precu, left precuneus; R Gyr Rectus, right gyrus rectus; R Med Orb Gyr, right medial orbital gyrus.

© 2023 Zhang X et al. *JAMA Network Open.*

eTable 2. Gray Matter Surface Morphological Alterations in Patients with OCD

Cluster	$p_{FWE\text{-}cluster}$	Number of vertices	Local maxima				Labels
			T	x (mm)	y (mm)	z (mm)	
thickness1	0.039	125	4.35	–43	–57	–21	L Fusi Gyr
gyrification1	0.008	205	3.73	25	49	32	R Sup Front Gyr
			3.68	25	50	20	R Sup Front Gyr
			3.25	33	49	15	R Mid Front Gyr
gyrification2	0.016	177	3.61	–10	1	40	L Mid Cing Gyr
			3.60	–7	15	33	L Mid Cing Gyr

Abbreviations: L Fusi Gyr, left fusiform gyrus; L Mid Cing Gyr, left middle cingulate gyrus; R Mid Front Gyr, right middle frontal gyrus; R Sup Front Gyr, right superior frontal gyrus.

© 2023 Zhang X et al. *JAMA Network Open.*

eTable 3. Performance Comparison of Brain Metrics for OCD Patient Identification

Input Variable \ Classifier Type		Logistic Regression				Linear Support Vector Machine			
		AUC	Accuracy (%)	Sensitivity	Specificity	AUC	Accuracy (%)	Sensitivity	Specificity
1	NDI @ R Occip Parie	0.8024	76.85	0.7963	0.7407	0.8034	76.39	0.8241	0.7037
2	WM-AD	0.7960	75.46	0.7593	0.7500	0.8006	75.93	0.7685	0.7500
3	WM-MD	0.7304	66.20	0.6389	0.6852	0.7396	66.20	0.6389	0.6852
4	GMV @ L Med Parie	0.5646	56.48	0.5926	0.5370	0.5876	56.48	0.5926	0.5370
5	GMV @ R Med Front	0.5415	56.48	0.5741	0.5556	0.5712	55.09	0.6204	0.4815
6	Thickness @ L Fusi Gyr	0.6419	59.26	0.5926	0.5926	0.6322	58.80	0.5926	0.5833
7	Gyrification @ L Mid Cing Gyr	0.6181	56.02	0.5648	0.5556	0.6326	56.02	0.5833	0.5370
8	Gyrification @ R Lat Front	0.6406	58.80	0.5741	0.6019	0.6446	61.11	0.5463	0.6759

Abbreviations: GM-NDI, gray matter neurite density index; GMV, gray matter volume; L Fusi Gyr, left fusiform gyrus; L Med Parie, left medial parietal regions; L Mid Cing Gyr, left middle cingulate gyrus; R Lat Front, right lateral frontal regions; R Med Front, right medial frontal regions; R Occip Parie, right occipitoparietal regions; WM-AD, white matter axial diffusivity; WM-MD, white matter mean diffusivity.

© 2023 Zhang X et al. *JAMA Network Open.*

eTable 4. Performance Comparison of Brain Metric Combinations for OCD Patient Identification

Classifier Type			Logistic Regression				Linear Support Vector Machine			
Image Modality	Metric Derivation	Input Variable(s)†	AUC	Accuracy (%)	Sensitivity	Specificity	AUC	Accuracy (%)	Sensitivity	Specificity
Diffusion	NODDI+GBSS	1	0.8024	76.85	0.7963	0.7407	0.8034	76.39	0.8241	0.7037
	DTI+TBSS	2 3	0.7923	74.07	0.7407	0.7407	0.7986	75.93	0.7685	0.7500
	combined	1 2 3	0.8554	77.78	0.7778	0.7778	0.8597	79.17	0.7963	0.7870
T1	VBM	4 5	0.5556	52.78	0.5463	0.5093	0.5803	54.63	0.5926	0.5000
	SBM	6 7 8	0.7337	68.06	0.6852	0.6759	0.7313	68.06	0.6852	0.6759
	combined	4 5 6 7 8	0.7393	68.98	0.7315	0.6481	0.7411	68.98	0.7407	0.6389
All combined			0.9052	81.48	0.8241	0.8056	0.9122	82.87	0.8241	0.8333

Abbreviations: GBSS, gray matter-based spatial statistics; SBM, surface-based morphometry; TBSS, tract-based spatial statistics; VBM, voxel-based morphometry.

† As claimed in eTable 3.

© 2023 Zhang X et al. *JAMA Network Open.*

eFigures

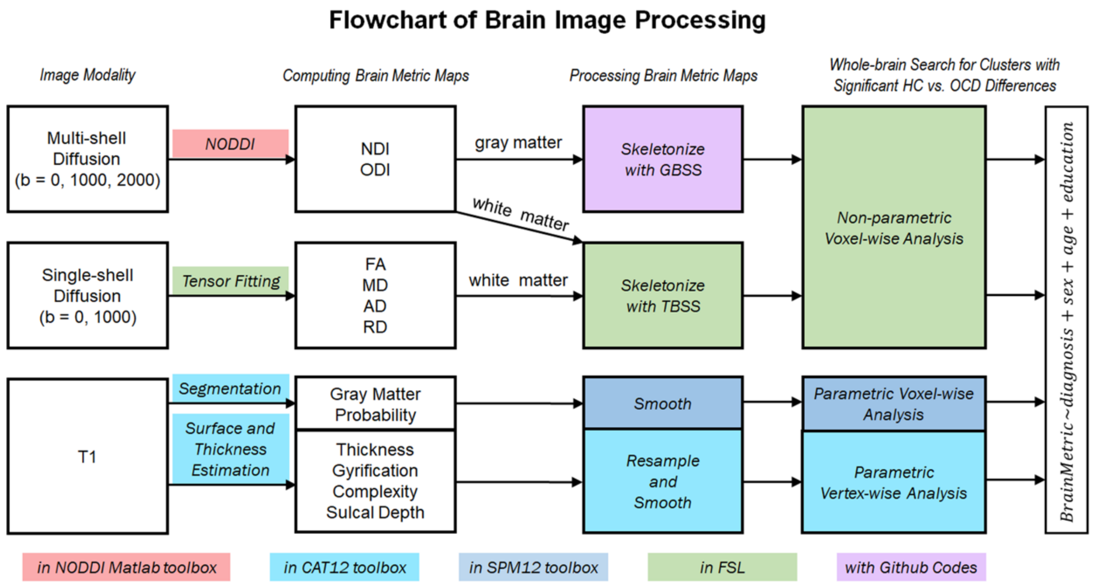

eFigure 1. The complete workflow for analyzing neuroimaging data. Our study employed a multi-modal approach by integrating various brain image modalities, including multi- and single-shell diffusion-weighted and T1-weighted brain images. The multi-shell diffusion-weighted images were subjected to neurite orientation dispersion and density imaging (NODDI), resulting in the extraction of parametric maps encompassing the neurite density index (NDI) and orientation dispersion index (ODI). Subsequently, the gray matter-based spatial statistics (GBSS) algorithm was employed to skeletonize the NDI and ODI maps for gray matter analysis, while the tract-based spatial statistics (TBSS) algorithm was used for white matter analysis. As for the single-shell diffusion-weighted images, tensor models were fitted to extract parametric maps, including fractional anisotropy (FA), mean diffusivity (MD), axial diffusivity (AD), and radial diffusivity (RD), which were then skeletonized using the TBSS algorithm. The T1-weighted brain images underwent voxel- and surface-based morphometry procedures within the Computational Anatomy Toolbox (CAT12) implemented in the Statistical Parametric Mapping (SPM12) toolbox. After preprocessing all the parameter maps, a general linear model was applied to identify clusters demonstrating significant main effects of OCD diagnosis while controlling for age, sex, and education. The diffusion-based metrics were analyzed using FSL, while the T1-based metrics were analyzed using SPM12.

© 2023 Zhang X et al. *JAMA Network Open.*

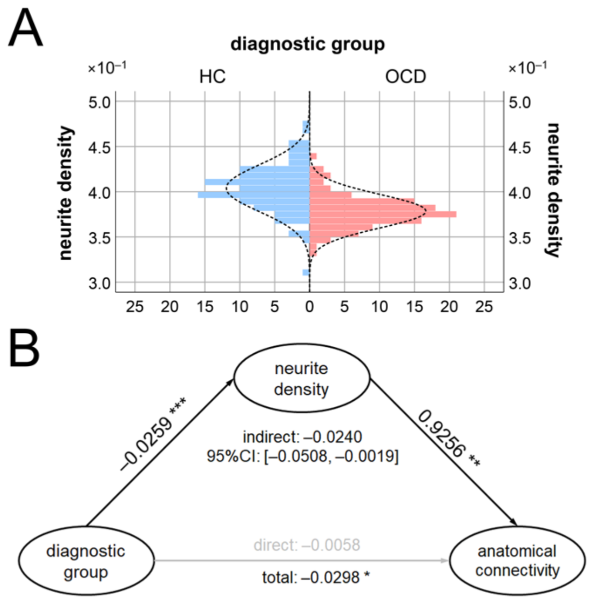

eFigure 2. Results of the NODDI-GBSS analysis. (A) The distribution of NDI values, averaged across voxels displaying a significant difference between the two groups, was compared between healthy participants and OCD patients. (B) The impairment in anatomical connectivity originating from the right lateral occipital and inferior parietal cortices towards other cortical regions in OCD was completely mediated by the local NDI. This mediation suggests that the deficit in dendritic density within the right occipitoparietal regions likely contributes to hindering their anatomical connections with other regions. (Abbreviations: GBSS, gray matter-based spatial statistics; HC, healthy control; NDI, neurite density index; NODDI, Neurite Orientation Dispersion and Density Imaging; OCD, obsessive-compulsive disorder. *** $p < 0.001$; ** $p < 0.01$; * $p < 0.05$)

© 2023 Zhang X et al. *JAMA Network Open*.

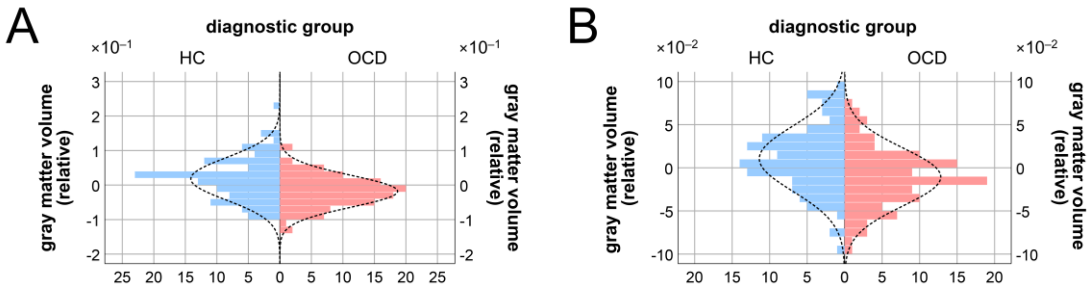

eFigure 3. Results of the Voxel-Based Morphometry. The distribution of gray matter volume values, averaged across voxels displaying a significant difference between the two groups in the left medial parietal (A) and the right medial frontal regions (B), was compared between healthy participants and OCD patients.

© 2023 Zhang X et al. *JAMA Network Open*.

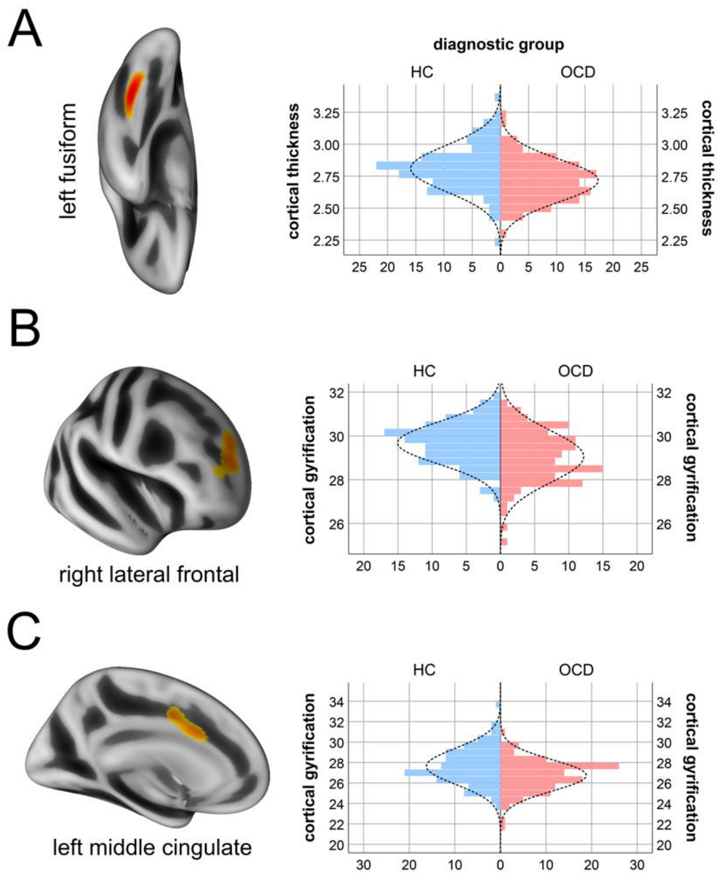

eFigure 4. Results of the Surface-Based Morphometries. Patients with OCD demonstrated significantly deficient cortical thickness in the left fusiform gyrus (A) and disrupted local gyrification in the right lateral frontal structures, i.e., the right superior and middle frontal gyri (B), and the left middle cingulate gyrus (C). The distribution of the cortical morphological metrics, averaged across voxels displaying a significant difference between the two groups was compared between healthy participants and OCD patients.

© 2023 Zhang X et al. *JAMA Network Open*.

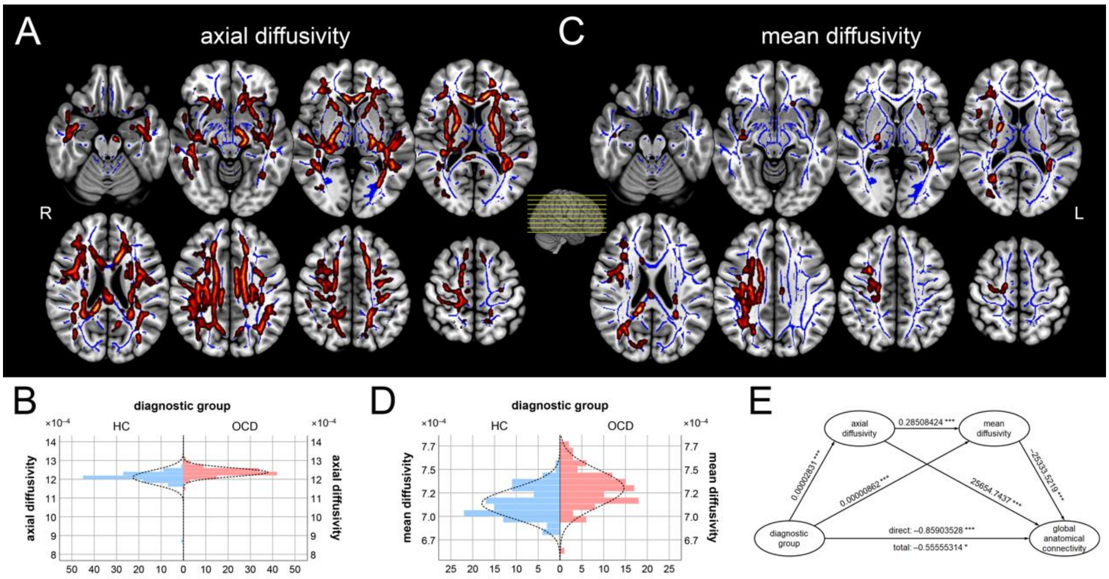

eFigure 5. Results of the white matter TBSS analysis. An extensive area of significantly greater white matter axial diffusivity (A) and mean diffusivity (B) was observed among patients with OCD compared to healthy participants. Alterations in axial diffusivity were present in both hemispheres, but those in mean diffusivity were lateralized to the right side. The distribution of diffusivity values, averaged across voxels displaying a significant difference between the two groups for AD (C) and MD (D), was compared between healthy participants and OCD patients. (E) The AD and MD in the identified regions partially mediated, in a serial manner, the impairment in the global strength of anatomical connectivity between all possible pairs of cortical regions. (Abbreviations: AD, axial diffusivity; L, left; MD, mean diffusivity; R, right; TBSS, Tract-Based Spatial Statistics. *** $p < 0.001$; * $p < 0.05$)

© 2023 Zhang X et al. *JAMA Network Open*.

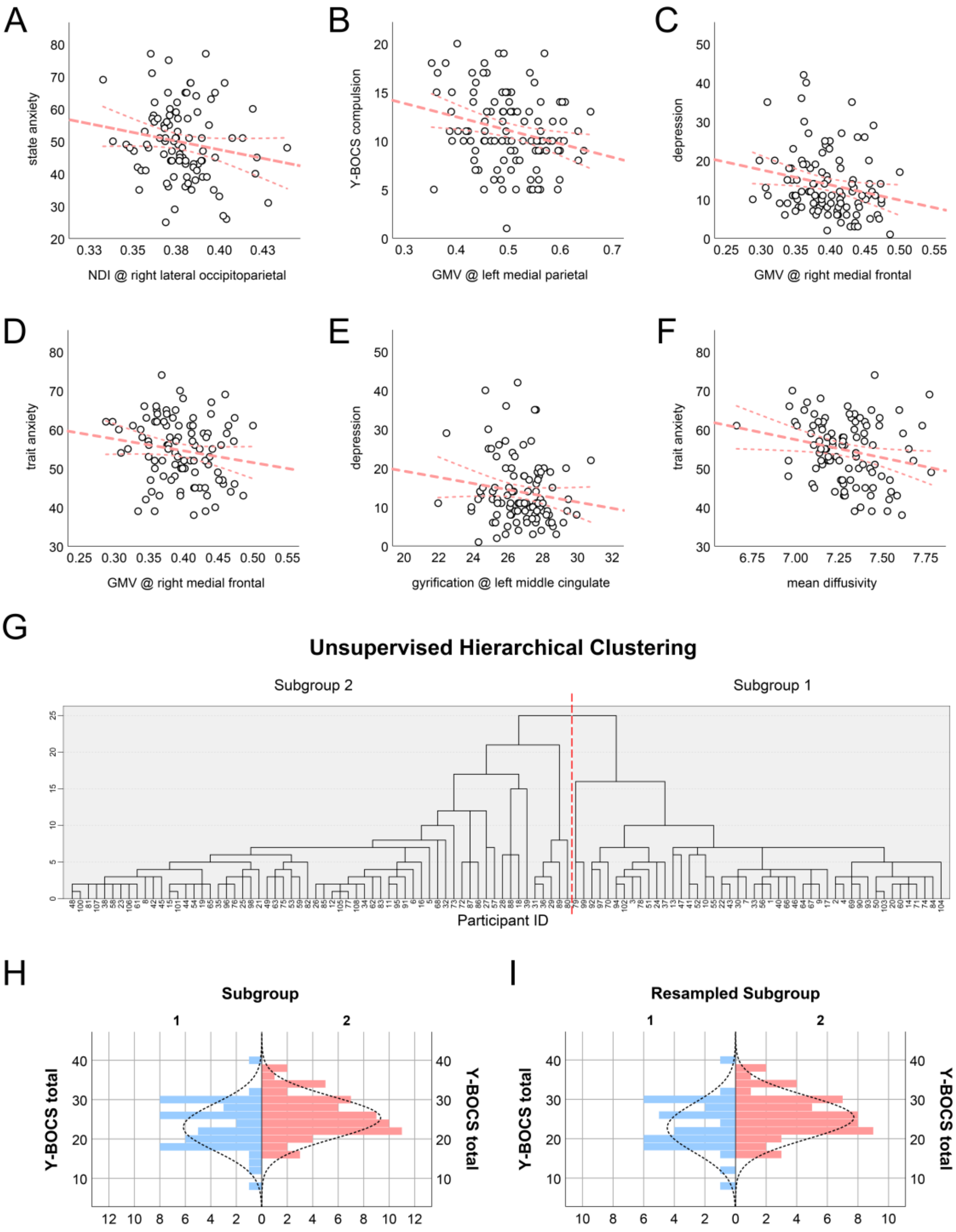

eFigure 6. Correlations between clinical symptoms and nodes of the pathological brain network in OCD patients. (A) The NDI at the right lateral occipitoparietal regions was negatively correlated with state anxiety. (B) The GMV at the left medial parietal regions was negatively correlated with the Y-BOCS compulsion score. (C, D) The GMV at the right medial frontal regions was negatively correlated with depression (C) and trait anxiety (D). (E) The gyrification at the left middle cingulate gyrus was negatively correlated with depression. (F) The white matter MD was negatively correlated with trait anxiety. (G) Unsupervised hierarchical clustering allowed for the classification of OCD patients into two subgroups based on the eight brain metrics. (H) The two subgroups did not significantly differ in age, education,

© 2023 Zhang X et al. *JAMA Network Open*.

or any clinical symptom except for the Y-BOCS total score. (G) This finding could be replicated in a subsample of OCD patients that were matched in terms of age, sex, and education. These findings suggest that the different nodes of the pathological brain network in OCD patients are associated with distinct clinical symptoms and provide relevant brain information for the assessment of the overall severity of OCD. (Abbreviations: Y-BOCS, Yale-Brown Obsessive Compulsive Scale)

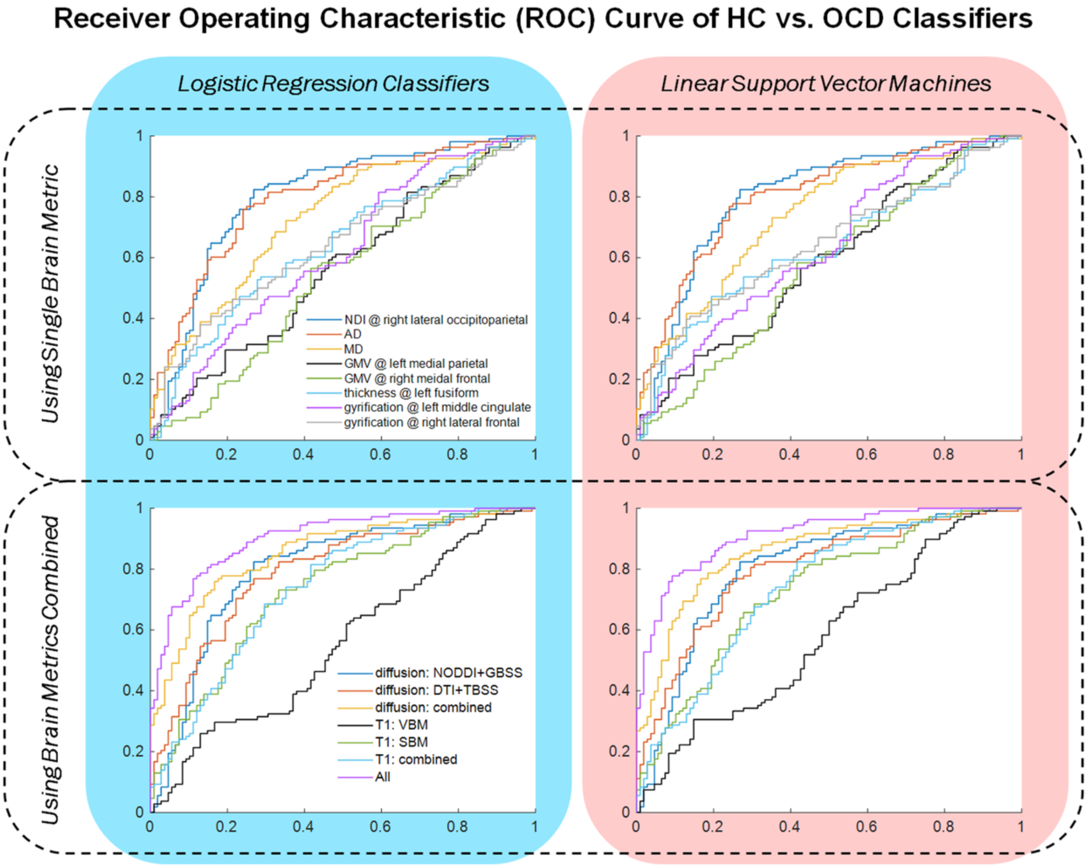

eFigure 7. Receiver operating characteristic (ROC) curves of HC vs. OCD classifiers based on different combinations of brain metrics. The classifier type tested here included logistic regression classifiers and linear support vector machines.

© 2023 Zhang X et al. *JAMA Network Open*.

eReferences

1. Li Q, Jiang L, Qiao K, et al. INCloud: integrated neuroimaging cloud for data collection, management, analysis and clinical translations. Gen Psychiatr. 2021;34(6):e100651.
2. Zhang H, Schneider T, Wheeler-Kingshott CA, Alexander DC. NODDI: practical in vivo neurite orientation dispersion and density imaging of the human brain. Neuroimage. 2012;61(4):1000-1016.
3. Nazeri A, Mulsant BH, Rajji TK, et al. Gray Matter Neuritic Microstructure Deficits in Schizophrenia and Bipolar Disorder. Biol Psychiatry. 2017;82(10):726-736.
4. Nazeri A, Chakravarty MM, Rotenberg DJ, et al. Functional consequences of neurite orientation dispersion and density in humans across the adult lifespan. J Neurosci. 2015;35(4):1753-1762.
5. Mori S, Zhang J. Principles of diffusion tensor imaging and its applications to basic neuroscience research. Neuron. 2006;51(5):527-539.
6. Hu Y, Li Q, Qiao K, et al. PhiPipe: A multi-modal MRI data processing pipeline with test-retest reliability and predicative validity assessments. Hum Brain Mapp. 2023;44(5):2062-2084.
7. Smith SM, Jenkinson M, Johansen-Berg H, et al. Tract-based spatial statistics: voxelwise analysis of multi-subject diffusion data. Neuroimage. 2006;31(4):1487-1505.
8. Bach M, Laun FB, Leemans A, et al. Methodological considerations on tract-based spatial statistics (TBSS). Neuroimage. 2014;100:358-369.
9. Ashburner J, Friston KJ. Voxel-based morphometry--the methods. Neuroimage. 2000;11(6 Pt 1):805-821.
10. Dahnke R, Yotter RA, Gaser C. Cortical thickness and central surface estimation. Neuroimage. 2013;65:336-348.
11. Luders E, Thompson PM, Narr KL, et al. A curvature-based approach to estimate local gyrification on the cortical surface. Neuroimage. 2006;29(4):1224-1230.
12. Yotter RA, Nenadic I, Ziegler G, Thompson PM, Gaser C. Local cortical surface complexity maps from spherical harmonic reconstructions. Neuroimage. 2011;56(3):961-973.
13. Van Essen DC. A Population-Average, Landmark- and Surface-based (PALS) atlas of human cerebral cortex. Neuroimage. 2005;28(3):635-662.
14. Smith SM, Nichols TE. Threshold-free cluster enhancement: addressing problems of smoothing, threshold dependence and localisation in cluster inference. Neuroimage. 2009;44(1):83-98.
15. Morris DM, Embleton KV, Parker GJ. Probabilistic fibre tracking: differentiation of connections from chance events. Neuroimage. 2008;42(4):1329-1339.
16. Desikan RS, Ségonne F, Fischl B, et al. An automated labeling system for subdividing the human cerebral cortex on MRI scans into gyral based regions of interest. Neuroimage. 2006;31(3):968-980.
17. MacKinnon DP, Fairchild AJ, Fritz MS. Mediation analysis. Annu Rev Psychol. 2007;58:593-614.
18. Hayes AF. Introduction to mediation, moderation, and conditional process analysis: A regression-based approach, 2nd edition: Guilford publications; 2017.

© 2023 Zhang X et al. *JAMA Network Open.*

19. Epskamp S, Fried EI. A tutorial on regularized partial correlation networks. Psychol Methods. 2018;23(4):617-634.
20. Epskamp S, Borsboom D, Fried EI. Estimating psychological networks and their accuracy: A tutorial paper. Behav Res Methods. 2018;50(1):195-212.
21. Murtagh F, Contreras P. Algorithms for hierarchical clustering: an overview. WIREs Data Mining and Knowledge Discovery. 2012;2(1):86-97.
22. Fithian W, Sun DL, Taylor JE. Optimal Inference After Model Selection. arXiv: Statistics Theory. 2014.
23. Kriegeskorte N, Lindquist MA, Nichols TE, Poldrack RA, Vul E. Everything you never wanted to know about circular analysis, but were afraid to ask. J Cereb Blood Flow Metab. 2010;30(9):1551-1557.
24. Boedhoe PS, Schmaal L, Abe Y, et al. Distinct Subcortical Volume Alterations in Pediatric and Adult OCD: A Worldwide Meta- and Mega-Analysis. Am J Psychiatry. 2017;174(1):60-69.
25. Boedhoe PSW, Schmaal L, Abe Y, et al. Cortical Abnormalities Associated With Pediatric and Adult Obsessive-Compulsive Disorder: Findings From the ENIGMA Obsessive-Compulsive Disorder Working Group. Am J Psychiatry. 2018;175(5):453-462.
26. Yun JY, Boedhoe PSW, Vriend C, et al. Brain structural covariance networks in obsessive-compulsive disorder: a graph analysis from the ENIGMA Consortium. Brain. 2020;143(2):684-700.

© 2023 Zhang X et al. *JAMA Network Open.*
