# Supplementary material for: Pathological Networking of Gray Matter Dendritic Density With Classic Brain Morphometries in OCD
Source: JAMA Netw Open. 2023 Nov 13;6(11):e2343208. doi: 10.1001/jamanetworkopen.2023.43208 (PMC10644219; doi:10.1001/jamanetworkopen.2023.43208)
Supplement: Supplement 2. — Data Sharing Statement [file jamanetwopen-e2343208-s002.pdf]

Data Sharing Statement

Zhang. Pathological Networking of Gray Matter Dendritic Density With Classic Brain Morphometries in OCD. *JAMA Netw Open*. Published November 13, 2023. doi:10.1001/jamanetworkopen.2023.43208

Data
Data available: No
